# Impact of mobile phones and wireless devices use on children and adolescents’ mental health: a systematic review

**DOI:** 10.1007/s00787-022-02012-8

**Published:** 2022-06-16

**Authors:** Braulio M. Girela-Serrano, Alexander D. V. Spiers, Liu Ruotong, Shivani Gangadia, Mireille B. Toledano, Martina Di Simplicio

**Affiliations:** 1 Division of Psychiatry, Department of Brain Sciences, Imperial College London, 7th Floor, Commonwealth Building, Du Cane Road, London, W12 0NN UK; 2 Westminster Children and Adolescents Mental Health Services, Central and North West London NHS Foundation Trust, London, W9 2NW UK; 3 MRC Centre for Environment and Health, School of Public Health, Faculty of Medicine, Imperial College London, St Mary’s Campus, Norfolk Place, London, W2 1PG UK; 4 NIHR Health Protection Research Unit On Chemical Radiation Threats and Hazards, School of Public Health, Faculty of Medicine, Imperial College London, St Mary’s Campus, Norfolk Place, London, W2 1PG UK; 5 Mohn Centre for Children’s Health and Wellbeing, School of Public Health, Faculty of Medicine, Imperial College London, St Mary’s Campus, Norfolk Place, London, W2 1PG UK

**Keywords:** Mental health, Child and adolescent, Mobile phone and wireless devices, Systematic review, Epidemiology

## Abstract

Growing use of mobiles phones (MP) and other wireless devices (WD) has raised concerns about their possible effects on children and adolescents’ wellbeing. Understanding whether these technologies affect children and adolescents’ mental health in positive or detrimental ways has become more urgent following further increase in use since the COVID-19 outbreak. To review the empirical evidence on associations between use of MP/WD and mental health in children and adolescents. A systematic review of literature was carried out on Medline, Embase and PsycINFO for studies published prior to July 15th 2019, PROSPERO ID: CRD42019146750. 25 observational studies published between January 1st 2011 and 2019 were reviewed (ten were cohort studies, 15 were cross-sectional). Overall estimated participant mean age and proportion female were 14.6 years and 47%, respectively. Substantial between-study heterogeneity in design and measurement of MP/WD usage and mental health outcomes limited our ability to infer general conclusions. Observed effects differed depending on time and type of MP/WD usage. We found suggestive but limited evidence that greater use of MP/WD may be associated with poorer mental health in children and adolescents. Risk of bias was rated as ‘high’ for 16 studies, ‘moderate’ for five studies and ‘low’ for four studies. More high-quality longitudinal studies and mechanistic research are needed to clarify the role of sleep and of type of MP/WD use (e.g. social media) on mental health trajectories in children and adolescents.

**Supplementary Information:**

The online version contains supplementary material available at 10.1007/s00787-022-02012-8.

## Introduction

Over the last ten years, the communication and information landscape has changed drastically with the development and rapid uptake of new portable devices such as smartphones or tablets, which are able to provide instant access to the internet anywhere. The likelihood of owning a smartphone increases with age, with market research reporting 83% of children in the UK aged 12–15 own a smartphone and 59% own a tablet. Up to 64% of children aged 12–15 have three or more devices of their own [1]. Alongside increased ownership rates, multifunctionality has expanded; a child’s phone may now enable internet browsing, games, applications, learning, online communication, and social networking.

The growing use of these technologies has raised concerns about how exposure patterns may affect children and adolescents’ wellbeing, as mental health disorders constitute one of the dominant health problems of this age group [2]. Increases in digital device usage have been hypothesized to be responsible for the secular trend of increasing internalizing symptoms, poorer wellbeing, and suicidal behaviours in adolescent populations [3]. It is reported that between 10–20% of children and adolescents suffer from a mental health problem globally [4, 5] and up to 50% of mental disorders emerge under the age of 15 [6]. A recent meta-analysis estimates the prevalence of any depressive disorder in children and adolescents is 2.6% (95% CI 1.7–3.9), and of any anxiety disorder is 6.5% (95% CI 4.7–9.1) [7]. Recent studies have shown that the usage of mobile devices in children and adolescents may be associated with depression [8–15], anxiety [8, 10, 15, 16] and with behavioural problems [17]. Particular patterns of smartphone-related behaviour, termed as ‘problematic smartphone use’ may be responsible for poor mental health associations [18].

Initially, research focussed on the physiological aspects of exposure to mobile phones or wireless devices (MP/WD) that use radiofrequency electromagnetic fields (RF-EMF). The Stewart Report identified that children and adolescents may be especially susceptible to exposure due to their developing nervous systems, greater average RF deposition in the brain compared with adults, and a longer lifetime of exposure [19]. It is still unclear whether exposure to RF-EMF from MP/WD can affect cognitive and emotional development in children and adolescents [20].

However, health effects of MP/WD on children and adolescents could also stem from psychological, social and behavioural factors related to their use. Adolescence is a dynamic phase of social and emotional development characterised by a change in the intensity and quality of communications among peers [21]. Adolescents have a constant need to interact and to be acknowledged by others, so that they can define their role and status in the peer group [22]. This distinctive pattern of socialization contributes to and is reflected by the pervasive use of social media embedded in MP/WD at this stage of life and research so far has focussed on this aspect.

Physiologically, adolescence is characterized by a delay in bedtime and a decrease in length of sleep with age [23], and sleep deficits are highly prevalent [24]. Given the pivotal role of sleep in adolescents’ health and development, research has investigated the associations between bedtime use of MP/WD, sleep disturbance and poor mental health outcomes. Studies to date report growing evidence of the detrimental impact of these technologies on sleep, although the specific relationship with mental health remains to be fully understood [25], including potential mechanisms such as (1) displacement of sleep by directly interrupting sleep time [26], (2) impact on circadian rhythm due to exposure to blue and bright light from screens [27] and (3) sleep disturbance due to the content of messages received pre-bedtime [28].

The complex relationship between factors including (but not limited to) exposure to RF-EMF, light from screens, engagement with internet or social media content, peer communication and their physiological and psychological consequences represents a challenge to determining definitive associations of interest between children and adolescents’ MP/WD use and mental health. This research field has evolved through different theoretical approaches and become the centre of media interest. However, previous reviews have either focussed on the psychological or behavioural aspects [29], or specifically on RF-EMF exposures for MP only [30, 31], and overlooked key information on confounders, such as socio-demographic factors. It is important when synthesizing these findings that all aspects of MP/WD use are considered. For example, mobile phone use is related to exposures hypothesized to have psychological effects (e.g., RF-EMF, screen-light), but these often occur simultaneously with changes of behaviour (e.g., reduced sleep, physical activity). Furthermore, different purposes of use may have different levels and temporal patterns of usage. Disentangling these effects often requires complex, tailored study-designs with advanced exposure measurement tools, and discussion of these issues with respect to MP/WD use and mental health is often missing. An assessment of the methodological quality of the available evidence to date could direct future research, policy and health recommendations around children and adolescents’ use of MP/WD. This evidence synthesis is also much needed now that digital tools for mental health hold the promise to overcome barriers to access support [32]. As the current COVID-19 pandemic has further accelerated the move towards a “digital mental health revolution”, it is crucial to identify if and under which conditions MP/WD use may be detrimental.

Our aims are to undertake a systematic review and appraisal of the evidence with a primary objective of assessing the relationship between duration or frequency of MP/WD use and children and adolescents’ mental health through synthesis of findings from individual quantitative observational studies conducting inferential analysis on this relationship. We define our exposure as any mobile or portable technologies that use RF-EMF to connect with the internet, cellular network, or cordless base station. This includes mobile phones, tablets and smartphones. Studies investigating only the use of devices that are not wireless (e.g. TV) or handheld in the same manner as tablets and phones (e.g. laptops) were excluded.

Secondary objectives are to synthesise findings on whether:

1. Impact on mental health is influenced by the temporal pattern (e.g. bedtime)
2. Different modes of use (e.g. calls, social media, instant messaging) have distinct effects on mental health
3. Impact on mental health differs for specific outcomes, in particular: internalizing symptoms (e.g. anxiety, depression, suicidal ideation/self-harm), externalizing symptoms (e.g. attention, concentration) and general wellbeing.

## Methods

### Search strategy and selection criteria

**Fig. 1:**
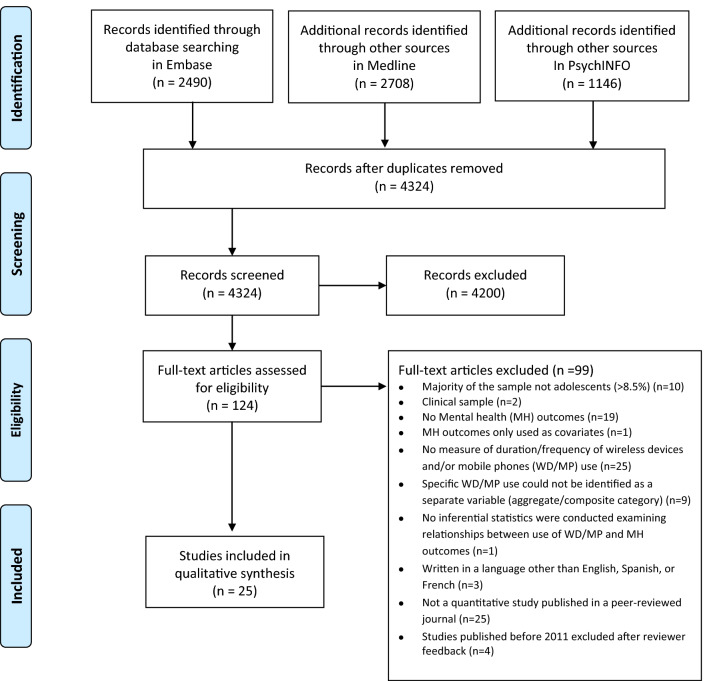
PRISMA Flowchart

This review was written in accordance with PRISMA statement recommendations (see Supplementary Material Table S1 for PRISMA checklist) [33] and was prospectively registered on PROSPERO (CRD42019146750) [34]. Relevant published articles were identified using tailored electronic searches developed with experts on MP/WD exposure and mental health (see Supplementary Material Table S2 for search terms list where we outline examples of exposures and mental health outcomes in detail). We originally searched Medline, Embase and PsycINFO using OVID interface for all studies published prior to July 15th 2019 (see PRISMA Flowchart Fig. 1). Both published and unpublished studies with abstracts and full texts in English, Spanish and French were searched. BGS and AS completed backward and forward citation tracking of included studies. Any inconsistencies between selected studies were resolved by discussing this with a third author (MDS).

Each study identified in the search was evaluated against the following predetermined criteria:

- Population: Studies examining children or adolescent populations where at least 70% of participants are aged 18 years or under.
- Exposure: Studies measuring daily or weekly duration or frequency of mobile phone or wireless device use (devices can include smartphones, cordless phones, tablets e.g., iPad).
- Outcomes: Studies that report a standardized and/or quantifiable measure (i.e., administered in a consistent manner across subjects) of mental health symptoms or psychopathology prevalence, which we define as to include: measures of internalizing symptoms and disorders (e.g. anxiety, depression, suicidal ideation/self-harm), externalizing symptoms and disorders (e.g. attention, and conduct disorders), and well-being measures (e.g. measures of self-esteem, health-related quality of life) among children and adolescents.
- Published in a peer-reviewed journal in English, Spanish or French.
- Reported inferential statistics describing cross-sectional or longitudinal associations between MP/WD usage and mental health outcomes.

Studies were excluded if: (1) specific wireless device use could not be identified as a separate variable (i.e., the main independent variable in the statistical model is a composite such as “digital media use”, “screen time”); (2) only clinical populations; (3) only investigated: physical health (e.g.: headaches, fingers/neck pain), somatic symptoms, cognitive functions (attention, memory), safety (driving, related accidents), relational consequences (relationships, physical fitness, worse academic performance, sexual behaviour (sexting), cyberbullying, sleep habits, personality, study assessment or intervention of substance use/addiction, specific apps, smartphone and social media loss, reviews or qualitative studies. (4) Case studies, opinion pieces, editorials, comments, news, letters and not available in full text. After reviewer feedback, we excluded all articles published before January 1st 2011 as MP/WD devices used before this period are unlikely have the same interactivity of devices used at the time of search.

### Data extraction and quality assessments

We (BGS, AS, ER, SG) extracted the data using a standard data extraction form (data extraction started on Aug 20, 2019). Data was verified by a second author, and then checked for statistical accuracy (AS or BGS). We chose to extract the estimands of associations from the final covariate-adjusted model specified by each group of study author, as not every iteration of the models was available to us. For transparency, the adjustment factors can be viewed clearly in the column second to the right of Tables 1, 2, 3, 4.

**Table 1:** General use of mobile phones and/or wireless devices: longitudinal findings

Study	Sample size (*n*), study design	Age group, gender distribution	Mobile phone & wireless devices measure	Mental health measure	Results	Covariates controlled for	Quality assessment
Babic, et al. [55] Australia	*N* = 322 Baseline: 2014 6-months follow-up Cohort: Switch-off 4 Healthy Minds	Mean age: 14.4 (SD = 0.6 years) 65.5% female	Daily duration of tablet and Mobile Phone (MP)	Internalising & Externalising: - Total Strengths and Difficulties Questionnaire (SDQ) Wellbeing: - Marsh’s Physical Self-Description Questionnaire - Flourishing Scale	Changes in duration of tablet/MP use (*β* = – 0.18, *p* < 0.001) were negatively associated with physical self-concept, but not associated with psychological well-being (*β* = 0.008, SE = 0.005, *p* = 0.078) or with total difficulties score from SDQ (*p* = 0.405)	Sex, Socioeconomic status (SES), body mass index (BMI), physical activity, baseline Mental Health (MH)	Moderate risk of bias
Bae [57] South Korea	*N* = 2110 Baseline: 2014 1-year and 2-year follow-up	Mean age: 10.98 [SD = 0.18] 48.5% female	Frequency of smartphone use for communication only (SUFC) purposes	Wellbeing: - Subjective Wellbeing (SWB) bespoke scale	Higher total scores for SUFC were associated with higher SWB scores (Cronbach’s ⍺ between 0.71 and 0.73). LGM analysis showed initial SUFC was associated with initial SWB (*β* = 0.50, *p* < 0.01), but change in SUFC over time had no significant direct effect on SWB intercept or slope A bootstrap test revealed that Social Capital mediated the relationship between SUFC and SWB; 31% CI = [0.258–0.361] by the intercept and 47% by the slope	Social capital, baseline MH outcome	Moderate risk of bias
Bickham, et al. [9] United States	Total = 92 Baseline: 2009 1-year follow-up	Mean age: 14.0 (Range:12.56–15.94) 46.8% female	Daily duration of MP use on school days and weekends from ecological momentary assessment (EMA) and from time use diaries	Internalising: - Depressive symptoms: Beck Depression Inventory (BDI) without suicidality items	Significant longitudinal positive association between depressive symptoms and “moments” of MP use assessed by EMA at 1-year follow-up (*β* = 0.220, *p* = 0.02), but not with time use diaries reported or self-reported duration MP use	Sex, ethnicity, parental education, and media use, baseline MH	Low risk of bias
George, et al. [45] USA	*N* = 151 Baseline: 2012 18-month follow-up Cohort: miLife Study	Mean age: 13.1 (SD = 0.91) 48% females	Daily duration of MP texting No. text messages sent by MP assessed using EMA	Externalising: - Conduct problem symptoms: bespoke dichotomous 6 items scale based on DSM–IV Conduct Disorder (CD) symptoms and modified items from the Child Behaviour Checklist	Average number of text messages sent reported predicted increases in conduct problems at 18-month follow-up assessment (*β* = 0.06, 95% CI = [0.02, 0.11], *p* = 0.007)	Baseline MH	Moderate risk of bias
Khouja, et al. [56] UK	*N* = 1869 Baseline: 2007 to 2009 2-years Follow up Cohort: ALSPAC	Mean age: 16 (SD = NA) 49% female	Daily duration of texting on MP on weekdays and weekends; usage categorized into three groups	Internalising: - Depressive symptoms: self-administered revised Clinical Interview Schedule (CIS-R) (ICD-10 diagnose) - Anxiety symptoms: self-administered CIS-R (ICD-10 diagnose) Categorised as: no anxiety/depression; symptoms but no diagnosis; and diagnosis	No association found between texting on weekdays and anxiety symptoms (1–2 h OR = 1.00 CI [0.84–1.20]; 3+ hours OR = 1.00 CI [0.77–1.30]) and no association found between texting on weekends and anxiety (1–2 h OR = 0.95 CI [0.79–1.14]; 3+ hours OR = 1.05, CI [0.81–1.35]) when fully adjusted for all confounders No association found between texting and symptoms of depression on weekdays (1–2 h OR = 1.05, CI [0.91–1.22]; 3+ hours OR = 1.00, CI[0.82–1.23]) or on weekends (1–2 h OR = 1.04, CI [0.89–1.21]; 3+ hours OR = 1.02, CI [0.85–1.24]) when fully adjusted for all confounders	Sex, SES, parenting/family, maternal age, maternal MH, bullying, Child IQ, physical activity, lifestyle	Low risk of bias
Liu, et al. [8] China	*N* = 3396 Baseline: 2017 8-month follow-up	Mean age: 18.3 (SD = 1.7) 19.1% female	Duration of MP use split into binary variable. Categorised into “high use” and “low use”	Internalising: - Depressive Symptoms: BDI - Anxious Symptoms: Self-Rating Anxiety Scale (SAS)	High MP use at baseline was associated with incident depressive and anxiety symptoms—Adjusted OR for incident depressive symptoms = 1.36 [1.04–1.79] (*p* < 0.05) and adjusted OR for incident anxiety symptoms = 1.34 [1.04–1.74] (*p* < 0.05) However, no association found between the longitudinal persistence of anxiety/depression and baseline high MP use Duration of follow-up MP use predicted by baseline anxious symptoms (*β* = 0.053, *p* < 0.001) and depressive symptoms (*β* = 0.058, *p* < 0.001)	Sex, age, SES, family/parenting, ethnicity, school type, overweight, sleep behaviours, TV/Internet, smoking, alcohol, Chronic medical conditions, educational attainment, lifestyle	High risk of bias
Poulain, et al. [50] Germany	*N* = 527 Baseline 2011 12-month follow-up Cohort: LIFE	Mean age: 3.81 (SD = 0.89)	Parent-reported duration of MP use with a 5-item Likert scale for frequency	Internalising & Externalising: - Parent-reported SDQ: Total Difficulties & all subscales excluding Prosocial subscale Subjects classified into normal and high-risk	Baseline MP use was associated with higher SDQ scores at follow-up for total difficulties (*β* = 2.21, CI = [0.62–3.79]), hyperactivity/inattention (*β* = 1.10, CI [0.31–1.88]) and conduct problems (*β* = 0.55, CI [0.01–1.10]). No association between baseline MP use and peer relationship or emotional problem subscale scores Baseline MP use associated with increased likelihood that child will be in high-risk category for total difficulties only OR = 3.25 CI [1.19–8.92]; baseline MP use not significantly associated with increase in likelihood of being in high-risk category for all subscales	Sex, age, SES, year of data acquisition, video use, game console use, computer internet use, baseline MH	Moderate risk of bias
Poulain, et al. [49] Germany	*N* = 814 Baseline 2011 1-year follow-up Cohort: LIFE	Mean age: 12.33 (SD = 1.67) 50.9% female	Daily duration of MP usage. Categorized into binary variable: “normal use” and “high use”	Internalising &Externalising: - SDQ: Total Difficulties & all subscales excluding Prosocial subscale Wellbeing: - Health-related quality of life (HRQoL): KIDSCREEN 27	No association found between baseline high MP use and follow-up SDQ (total difficulties score (*p* = 0.973), or any of the SDQ subscales Adolescents with high use of MP at baseline reported lower psychological WB at follow-up (*β* = – 0.07, CI = [– 0.13, – 0.01], *p* = 0.032)	Sex, age, SES, year of data acquisition; physical activity	Moderate risk of bias
Schoeni, et al. [48] Switzerland	Total = 425 Baseline 2012 1-year follow-up HERMES cohort	Mean Age: 15.0 (SD: 0.79) 59.8% female	Duration of MP calls, data traffic (e.g. streaming) and number of text messages sent/received (SMS, WhatsApp etc.) RF-EMF dose measures: near-field component (MP, cordless phones, laptop/tablet connected to WLAN) + far-field component (radio and TV broadcast transmitters)	Externalizing Symptoms - Concentration difficulties: 4-point Likert scale for symptom severity converted to binary variable	Symptoms of concentration difficulties associated with self-reported text messages sent per day (OR = 2.57, CI [1.35–4.89]), and operator data-determined text messages sent (OR = 0.97, CI [0.83–1.13]) Symptoms of concentration difficulties significantly associated with: self-reported duration of data traffic on MP (OR = 1.97, CI [1.14–3.40]) but not with operator data-derived volume of data traffic (OR = 1.20, CI [0.99–1.44]) Concentration difficulties was also associated with operator data-derived duration of mobile phone calls (OR = 1.21, CI [1.03–1.44]), however no significant association was found between concentration difficulties and self-reported duration of mobile phone calls (OR = 1.14, CI [0.93–1.38]) The same symptoms were significantly associated with self-reported duration of cordless phone calls (OR = 1.64 CI [1.27–2.12]) Symptoms of concentration difficulties were associated with cumulative RF-EMF whole-body dose from usage of wireless devices from operator data (OR = 1.13, CI [0.98–1.31]), but not from self-report usage (OR = 1.10 CI [0.88–1.38]). Concentration difficulties were also not associated with cumulative RF-EMF dose to the brain self-reported data (OR = 1.02 CI [0.79–1.32]), nor from operator data (OR = 1.13 CI [0.98–1.31])	Sex, age, ethnicity, educational attainment, physical activity, alcohol, SES, change in body height	Low risk of bias

**Table 2:** Bedtime use of mobile phones and/or wireless devices: longitudinal findings

Study	Sample size (n), Study design	Age group, Gender distribution	Mobile phone & wireless devices measure	Mental Health measure	Findings of Interest	Covariates controlled for	Quality assessment
Vernon, et al. [60] Australia	*N* = 1101 Baseline 2010 3-years follow-up	Mean age: 13.5 (SD = NA) 57% female	Night-time MP use (text messages or phone calls) measured with a 6-point scale question	Internalising: - Depressed mood: 5 items measured with a 6-point Likert scale for frequency of symptoms Externalizing: - Externalizing behaviour: 7 items measured with a 8-point Likert scale for frequency of symptoms Wellbeing: - Self-Esteem: 3 items measured with a 6-point Likert scale for frequency of symptoms - Coping: One item from the NEO Personality Inventory–Revised	Initial night-time MP use directly associated with initial depressed mood, externalizing behaviours, decreased self-esteem and not with coping ability. Changes in night-time MP use significantly associated with subsequent changes self-esteem, externalizing behaviour and coping, but not depressed mood Indirect total effect size ratio from bootstrapped mediation analysis estimated that all well-being outcomes were mediated by both initial sleep problems and the change in sleep problems. Proportion of mediated effect for depressed mood was 77% mediated by initial sleep problems and 73% mediated by changes in sleep. Externalizing behaviour was 33% mediated by initial sleep as well as changes in sleep. Both coping and self-esteem had a high proportion of mediated effect by initial sleep (91 and 83% respectively) and by changes in sleep (50 and 60% respectively)	Sex, age, SES, sleep behaviours	Low risk of bias

**Table 3:** General use of mobile phones and/or wireless devices: cross-sectional findings

Study	Sample characteristics	Mobile phone & wireless devices measure	Mental health measure	Findings of interest	Covariates controlled for	Quality assessment
Calpbinici and Arslan [15] Turkey	Total: 426 Mean age: 16.05 (SD = 1.26) 49.5% female	- Daily duration of MP calls categorized into “none, ≤ 1 h, > 1 h” - Purpose of MP usage (talking/messaging, social media)	Internalising: - Anxiety and depression symptoms: subscales from Brief Symptom Inventory (BSI) Externalising: - Hostility: subscale from BSI Wellbeing: - Negative Self‐esteem subscale from BSI	No significant difference between groups with daily speaking duration with regards any of the BSI subscales Adolescents who used MP more social media had significantly higher BSI mean for every subscale (*p* < .001)	None	High risk of bias
Foerster and Röösli [46] Switzerland	Total: 412 Mean age: 14.1 (Range 10.4–17.0) 56.9% female	Specific use of MP during weekend and weekdays: calls (minutes/day), text messages (f/day), online on MP (m/day), social networking. Categorised into “low, medium, high”	Wellbeing: - Health-Related Quality of Life (HRQoL): KIDSCREEN-52	Latent class analysis of MP use, general media use, and MPPUS-10 scores identified 5 distinct classes: Low Use, Medium Use, Gaming, Call Preference and High Social Use Significant difference in HRQoL found across groups in Physical Wellbeing (*p* = 0.002), Moods & Emotions (*p* < 0.001), Self-Perception (*p* > 0.001), Self-perception (*p* < 0.001), Parent Relations & Home Life (*p* < 0.001), Social Support & Peers (*p* < 0.001) & School Environment (*p* < 0.007). For the other four scales (Psychological Wellbeing, Autonomy, Financial Resources, Social Acceptance) no significant differences between the classes were found	Sex, age, SES, ethnicity, educational attainment	High risk of bias
George, et al. [45] USA (cross-sectional findings only)	Total 151 Mean age: 13.1 (SD = 0.91) 48% female	Hours spent texting and text messages sent by MP using ecological momentary assessment (EMA)	Internalising: - Depressive Symptoms: 5 dichotomous items from the Beck Depression Inventory (BDI) - Anxiety symptoms: 4 dichotomous items from the Multidimensional Anxiety Scale for Children Externalising: - ADHD symptoms: 4 dichotomous items adapted from the DSM–IV symptom checklist - CD symptoms: 6 dichotomous items from DSM–IV and Child Behaviour Checklist *all measures modified for EMA	Adolescents reported fewer anxiety (*p* = 0.007; incident rate ratio (IRR) = 0.99) and fewer depression symptoms (*p* = 0.006, IRR = 0.99) on days where they sent more text messages Association found between time on mobile phone texting and reduced anxiety symptoms, (*b* = 0.05 95% CI [0.09, 0.01] *p* < 0.05), but with increased risk of conduct disorder (*b* = 0.22 95% CI [0.10, 0.33] *p* < 0.001) and with increased risk of ADHD (*b* = 0.10 95% CI [0.04, 0.18] *p* < 0.01) No association found between duration of texting and depressive symptoms	MH baseline	High risk of bias
Guxens, et al. [52] Netherlands	Total: 3102 Mean age: 5 Female: not reported	Parent-reported frequency of MP and cordless phone calls	Internalising & Externalising: - Mother & teacher-reported Strengths & Difficulties Questionnaire (SDQ): Total Difficulties & all subscales. Subjects categorized in normal, borderline, and abnormal	MP use for category < 1call/week had lower odds of teacher-reported total difficulties SDQ borderline/abnormal score when compared with no MP use (OR = 0.67, CI = [0.47–0.95]), but not for mother-reported total difficulties MP use for category < 1call/week had smaller risk of mother-reported peer relationship SDQ borderline/abnormal score than those with no use (OR = 0.61, CI = [0.42–0.91]) Children with cordless phone at home had lower odds of teacher-reported borderline/abnormal prosocial behaviour (OR = 0.68, CI = [0.48–0.97]) and lower odds of mother-reported peer relationship problems (OR = 0.61, 95% CI = [0.39–0.96]) No other teacher- or mother-reported SDQ subscales was significantly associated with cordless/MP phone calls Null result found when testing for trend between cordless/MP use and SDQ total difficulties score	Sex, age, SES, family/parenting, Maternal factors (education, ethnicity, BMI tobacco, alcohol, MH, attachment)	High risk of bias
Hosokawa and Katsura [51] Japan	Total: 1642 Mean age: 6.88 (SD = 0.35 years) Female:48.8%	Parent-reported average typical daily duration of smartphone and tablet use. Categorized into “regular and non-regular users”	Internalising & Externalising: - Parent-reported SDQ. Subjects categorized into normal, borderline, abnormal	Regular mobile device uses significantly associated with higher externalizing problems, specifically conduct problems (OR: 1.77, 95% CI: [1.03 ± 3.04], *p* < .05) and hyperactivity /inattention (OR: 1.82, 95% CI: [1.15 ± 2.87], *p* < .01) Regular mobile device uses not significantly associated with internalizing problems	Sex, SES, parenting/family, baseline MH	High risk of bias
Ikeda and Nakamura [10] Japan	Total: 2698 First-years: 45.9% Second-years: 48.9 Third-years: 5.2% Female: 62.3%	Average mean duration of MP use per week and per weekday and then stratified by quartiles	Internalising: - 4 subcomponents (“Tension and excitement,” “Refreshing mood” “Depressed mood”, “Anxious mood.”) from the Mood Inventory	Increased duration of MP use per week is associated with lower psychological mood, for tension and excitement (*p* < 0.001), and particularly depressed mood (*p* < 0.009)**.** For males, total scores for ‘‘Depressed mood,’’ and ‘‘Tension and excitement,’’of the highest quartile of weekly MP use were significantly higher than for other quartiles (*p* < 0.05) No association between MP use and mood scores was found in female subgroup	Sex, age, school type, physical activity, previous MP-use, sleep behaviours	High risk of bias
Mortazavi, et al. [16] Iran	Total: 469 Mean age: 11 (SD = 2.33) 49.89% female	Daily average duration of MP use for talking. Categorized into 4 groups: “No Use, Less than 10 min, 11–30 min, more than 30 min”	Internalising: - Anxiety: 1 item Externalising: - Concentration problems:1 item - Attention problems: 1 item All items measured by 4-point Likert scale for frequency of symptoms	Association between the duration of MP use when talking and self-reported symptoms of concentration problems (*p* < 0.001), attention problems (*p* < 0.001) and anxiety (*p* < 0.001) were found in students who had used MPs compared to those who had never used phones	None	High risk of bias
Nishida, et al. [12] Japan	Total: 295 Mean age: 16.2 (SD = 0.9) 41.4% female	Daily duration of type of smartphone use including email, social networking sites, online chat, internet search and watching videos	Internalising: - Depression: - Centre for Epidemiological Studies Depression (CES-D). Classified as depressed if score > 16	No association between general smartphone use and depression among male students OR = 1.09, CI = [0.65–1.82] or females student OR = 1.58, CI = [0.95–2.63]) Stratified analysis by gender showed Increased duration of online chat (OR = 1.7, CI = [1.18–2.56]), and SNS (OR = 1.41, 95% CI = [1.04–1.92]) using smartphone was associated with depression among female students, but not in males (OR = 1.09, 95% CI = [0.65–1.82]) *p*:0.737	Sex, age, educational attainment, lifestyle, sleep behaviours, parenting/family	High risk of bias
Przybylski and Weinstein [59] England	Total: 120,115 Mean age:15 (SD not reported) Female: Not reported	Daily duration of smartphones for social networking and chatting during free time on weekdays and weekend with categories	Well-being: - Psychological Well-being: Warwick-Edinburgh Mental Well-Being Scale	Inverted-U-shape relationship between digital-screen time, smartphones and mental WB. Moderate engagement not harmful and may be advantageous. Optimum / extremum for weekday smartphone use is 1 h 57 min; optimum/extremum for weekend smartphone use is 4 h 10 min Effect size of weekday smartphone use on WB above extremum is *b* = – 0.53, 95% CI [– 0.56, – 0.49] Cohen’s *d* = – *0.20* Effect size of weekend smartphone use on WB above extremum is *b* = – 0.83, 95% CI [– 0.94, – 0.74] Cohen’s *d* = – *0.14*	Sex, SES, ethnicity, technology access	High risk of bias
Redmayne, et al. [14] New Zealand	Total: 373 Mean Age: 12.3 (Range: 10.4–13.7) 44.2% female	Frequency (number and duration) of cordless and MP use and type of MP headset. Cordless phone operating frequency, modulation system/approach	Internalising symptoms: - Depressive symptoms: 1 item from the Health Behaviour in School-aged Children checklist measured by a 4-point Likert scale for frequency of symptoms	Authors were not able to fit valid models with exposure variables of duration of cordless phones and MP calls, i.e. they could not determine if duration or frequency of use of wireless devices were associated with depressive symptoms Use of wired (OR = 0.90 [0.51–1.58]) or wireless (OR = 2.04 [1.09–3.82]) MP headsets was associated with frequency of depressive symptoms. Cordless phone frequency also associated with depressive symptoms, but only for frequencies ≤ 900 MHz (OR = 2.40 [1.15–5.02])	Sex, age, SES, woken by phone, illness, earpiece, headset, TV in bedroom	High risk of bias
Roser, et al. [47] Switzerland	Total: 412 Mean age: 14.0 (Range 12.1–17.0) 61.4% female Cohort: HERMES	Self-report: - Frequency of outgoing/incoming calls - Frequency of outgoing text messages and duration of data traffic Data from MP operators: - Frequency of outgoing/incoming calls - Outgoing SMS and the volume of data traffic over previous 6 months	Internalising &Externalising: - Parent & self-reported SDQ: Well-being: - HRQoL: KIDSCREEN-52	Ten-point increase in MPPUS-10 score was positively associated with *β* = 0.96 total difficulty score (95% CI [0.58, 1.35] *p* < 0.001). Positive association also found with hyperactivity (*β* = 0.42, 95% CI [0.26, 0.57]), conduct problems (*β* = 0.30, 95% CI [0.19, 0.41]) emotional symptoms (*β* = 0.17, 95% CI [0.02, 0.32]). Prosocial behaviour was significantly negatively associated with MPPUS-10 (*β* = – 0.14, 95% CI [– 0.25, – 0.04]) Six out of the ten HRQOL dimensions were significantly decreased (Moods and emotions, Self-perception, Autonomy, Parent relations and home life, Financial resources and School environment) in adolescents with higher MPPUS-10 score	Sex, age, SES (educational level of parents), school level, nationality, self-reported freq of text messages sent	High risk of bias
Tamura, et al. [11] Japan	Total: 295 Mean age: 16.2 (SD = 0.9) 41.4% female	Daily duration of type of smartphone use including email, social networking sites, online chat, internet search and watching videos	Internalising: - Depression: CES-D. Classified as depressed if score > 16	Mobile phone use of ≥ 2 h per day for social network services (OR: 3.63, 95% CI [1.20–10.98]) and online chats (OR: 3.14, 95% CI [1.42–6.95]), was associated with a higher risk of depression, even when adjusting for sleep duration	Sex, age, school type, lifestyle, sleep behaviours, lifestyle, parenting/family	High risk of bias

**Table 4:** Bedtime use of mobile phones and/or wireless devices: cross-sectional findings

Study	Sample characteristics	Mobile phone & wireless devices measure	Mental health measure	Key findings	Covariates controlled for	Quality assessment
Lemola, et al. [61] Switzerland	Total: 362 Mean age: 14.8 (SD = 1.3) 44.75% female	- Electronic media use in bed before sleep for the following activities: talking on the phone/texting in chat rooms or surfing the Internet. Measured with a 5-point Likert scale - MP type ownership (smartphone or phone with basic features)	Internalising: - Depressive symptoms: 6 items from the ADS-K (German version of CES-D)	Adolescents with smartphones are not significantly more likely to have depressive symptoms than those that have mobile phones with basic features only (*F* = 0.79, *p* = 0.37) Depressive symptoms are significantly correlated with calling/text messaging in bed (*r* = 0.18, *p* < 0.001) and with being online (Facebook, Chat etc.) in bed (*r* = 0.22, *p* < 0.001) Electronic media use in bed before sleep was related to higher levels of depressive symptoms (*β* = 0.26, *t* = 4.84, *p* < 0.001), shorter sleep on weekday nights (*β* = – 0.29, *t* = – 6.00, *p* < 0.001) and greater sleep difficulties (*β* = .21, *t* = 3.91, *p* < 0.001) The presence of sleep difficulties partially mediates relationship between electronic media use at night and depressive symptoms (from *β* = 0.26, *t* = 4.84, Δ*R*^2^ = 0.060; *p* < 0.001 to *β* = 0.17, *t* = 3.49, Δ*R*^2^ = 0.026; *p* < 0.001)	Sex, age, sleep behaviours	High risk of bias
Mei, et al. [58] China	Total: 3020 7th–11th grade students 49.21% female	- Frequency of MP use before bedtime (never/sometimes/often) - Daily duration of bedtime MP use before sleep - Which mode of MP was most time-consuming function out of following options: call, surfing the Internet, texting - Daily duration of above most time-consuming function	Internalising: - Depression: BDI-II - Anxiety: SAS	MP use before sleep associated with more depressive symptoms (*β* = 0.89, *p* < 0.001) and anxiety symptoms (*β* = 1.22, *p* < 0.001) Reduced sleep duration partially mediated this association (*β* reduced to 0.16 and 0.22 for anxiety and depression respectively, *p* < 0.001) Adolescents who often used MP before sleep, sleep duration was significantly shorter and sleep onset latency was significantly longer than adolescents who either sometimes or never used MP except to study (*p* < 0.05)	Age, sex, SES, BMI, parenting/family, neighbourhood, health condition, educational attainment, academic stress, smoking, alcohol, snoring, sleep behaviours	High risk of bias
Mireku, et al. [54] UK	Total: 6616 Mean age: - Males: 12.1 (SD = 0.6) - Females: 12 (SD = 0.5) 48.8% female	Screen-based media devices (SMBD) of MP, tablet, eBook reader, laptop, within 1 h before sleep	Wellbeing: - HRQoL: KIDSCREEN-10	Adolescents who used MP during night-time reported lower HRQoL compared to those who did not use MP during night-time (OR = – 0.84, 95% CI [– 1.44, – 0.024] and use in a dark room was associated with even lower KIDSCREEN-10 score (*β* = – 1.18, 95% CI [– 1.85, – 0.52]) compared to no use Association found between use of at least one SBMD and lower HRQoL (OR = – 1.15, 95% CI = [– 1.82, – 0.48])	Sex, age, SES, ethnicity, school type, BMI, second-hand smoking, alcohol, caffeine in sensitivity analysis)	High risk of bias
Oshima, et al. [28] Japan	Total: 17,920 Mean age: - Early Adolescents: 13.7 (SD = 0.9) - Late Adolescents: 16.6 (SD = 0.9) 50.41% female	Frequency of MP use after lights out measured with a 3-point Likert scale	Internalising: - Suicidal feelings: 1 item measured with a 3-point Likert scale for agreement of symptoms - Self-harm behaviours over previous year: 1 dichotomous item; If Yes subjects asked to described method Wellbeing: - Psychological Wellbeing measured with General Health Questionnaire-12. If scored > 4 categorized as poor mental health	Early adolescents (EA) and late adolescents (LA) using mobiles phones after lights out ‘almost every day’ were more likely to have poorer mental health (EA: OR = 1.65; 95% CI [1.43–1.92]; *p* < 0.001, LA: OR = 1.54; 95% CI [1.38–1.72]; *p* < 0.001) compared to those who did not use their mobile phones after lights out Adolescents using mobile phones use after lights out also more likely to have more suicidal feelings (EA: OR = 1.62; 95% CI [1.31–1.99]; *p* < 0.001, LA: OR = 1.22; 95% CI 1.04–1.42; *p* = 0.014) and more likely to experience self-injury (EA: OR = 1.56; 95% CI [1.12–2.17]; *p* = 0.009, LA: OR = 1.75; 95% CI [1.33–2.29]; *p* < 0.001) compared to those who did not use their mobile phones after lights out	Age, sex, alcohol, drug use and sleep behaviours	High risk of bias

Authors of original papers were contacted to provide missing (subsample) data where necessary. AS and BGS both appraised each study independently for methodological quality and risk of bias using checklists adapted from the Newcastle–Ottawa Scale (NOS), originally designed to evaluate cohort studies [35], and considered a useful tool to assess risk of bias [36]. We used a customized checklist for cross-sectional studies, following an approach taken by previous systematic reviews of observational research [37, 38]. We also used the STROBE individual component checklist to critically appraise the aspects of reporting related to risk of bias, e.g. study design or sampling methods [39]. We defined the most important covariate adjustment factors as previous diagnosis of mental disorder or prior mental health and demographic confounders (sex, age, socioeconomic status (SES)) based on the Newcastle–Ottawa quality assessment Scale (NOS). We then categorized studies by quality and risk of bias based on accepted thresholds for converting the Newcastle–Ottawa scales to AHRQ standards [40]. A description of the conversion rules can be found in the footnotes to Table S6 and S7 in the Supplementary Material.

### Data synthesis

Given the high heterogeneity of the retrieved studies with regards to the primary explanatory variable of interest (MP and WD usage), the outcomes of interest (mental health), the objectives and the statistics used, statistical pooling was considered to be inappropriate and the quantitative data is synthesised narratively.

We classified studies by MP/WD exposure: (a) general MP/WD use (frequency/duration) and (b) bedtime MP/WD use; and by mental health outcomes: internalising symptoms, externalising symptoms and wellbeing. Children and adolescents’ emotional, behavioural and social difficulties are widely conceptualised in internalizing and externalizing symptoms groupings [41], endorsed by the DSM-V to provide directions in clinical and research settings [42]. We added a third category of wellbeing, to group scales measuring resilience, self-esteem, self-efficacy, optimism, life satisfaction, hopefulness etc., which are important indicators of how mental health is subjectively perceived and often valued by individuals above clinical symptoms [43, 44].

## Results

All retrieved studies meeting eligibility criteria (*N* = 25) were observational and investigated both genders. Ten (40%) employed a longitudinal design, while the remaining 15 (60%) had a cross-sectional design. One study [45] reported both cross-sectional and longitudinal findings. There were multiple studies drawing from the same population: three from the HERMES cohort [46–48], two from the LIFE cohort [49, 50] and two from the same sample of high-school students [11, 12].

The total number of research subjects was 164,284 who were aged between five and 21 years old. Most studies examined typically developing adolescents aged 8–18 years old. Three studies looked at young children aged 2–7 years old [50–52]. One study that included young people aged up to 21 years old was included in the review as ~ 70% of the samples met the ≤ 18-years old criteria [8].

Studies investigating associations of mental health outcomes with only aggregated screen time without device-specific measures were excluded from the review. All studies measured MP use. Three studies also investigated the effect of cordless phone usage [14, 17, 48, 52, 53]. Two studies also included specific measures of tablets [51, 54]; one study investigated other categories of WD including: eBook reader, laptop, portable media player and portable video game console [54]. Most studies used self-report questionnaires to assess MP/WD use: for example, asking participants to rate their daily or weekly use to best match an interval provided by the questionnaire [8–13, 15, 16, 46, 48, 53, 55, 56], or with ordinal scales of frequency [14, 28, 47, 57, 58]. Studies with young children instead used parent questionnaires [50–52]. Twenty studies reported MP/WD general use and five with bedtime use. Seven studies collected data of MP/WD usage on weekends and weekdays separately [9, 10, 45, 46, 55, 56, 59], with five of these reporting associations with mental health separately for weekday and weekends [9, 10, 55, 56, 59]. Twenty studies reported internalizing symptoms, 11 externalizing symptoms, and ten well-being measures.

Details on study aim, sample characteristics, MP/WD use, mental health outcomes and measures, and findings are summarised in Tables 1, 2, 3, 4.

### Quality assessment

The median and mean NOS scores of the longitudinal studies were 6 and 6.3 respectively. The median and mean scores for cross-sectional studies were 5 and 5.0 respectively. We converted each NOS Score for the 25 studies to AHRQ standards: risk of bias was rated as “high” for 16 studies, “moderate” for 5 studies and “low” for 4 studies. Risk of information bias was common as self-report measures were prevalent for outcome and exposure assessment. Additional factors contributing to high risk of bias included: risk of selection bias, attrition, and the absence of adjustment for confounding factors. All details regarding quality assessment, including summaries of risk of bias across studies, are reported in the Supplementary Material (Tables S4-S8).

### Main Research Findings

**Fig. 2:**
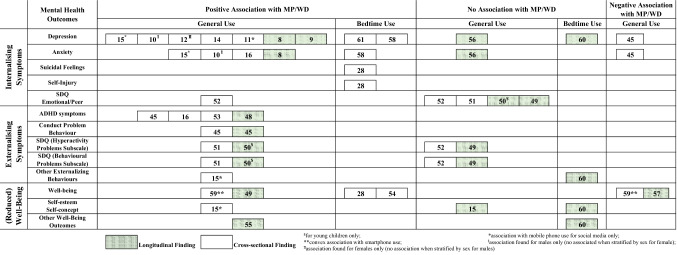
Harvest plot of associations between MP/WD usage and mental health outcomes among children and adolescents included in the systematic review. Numbers refer to study references as cited in the reference list. Two studies [46, 47] were excluded from this plot as they did not report direct inferential statistics between MP/WD and mental health

Findings are presented by exposure time (general or bedtime), design (longitudinal or cross-sectional) and outcome assessed (internalising symptoms, externalising symptoms and wellbeing). For each group of longitudinal findings, we report the AHRQ Quality Band (“high”, “moderate” or “low” below refer to risk of bias). Figure 2 categorises effects reported by direction of association with mental health outcome and by whether bedtime or daily aggregate MP/WD usage was investigated. All cross-sectional studies were rated as high risk of bias, so for brevity these are not reported in the text below. Unless otherwise stated, we describe associations adjusted for all confounding variables reported in each study (see Tables 1, 2, 3, 4 for details of covariates included in adjusted models).

#### General use of wireless devices

##### Longitudinal findings

Nine out of the 10 longitudinal studies included in this review examined associations between mental health outcomes and general use of MP/WD (Table 1).

*Internalising symptoms*: Two out of five studies (one low risk, one high) found a significant association between general use of MP/WD and measures of internalising symptoms. Bickham et al. [9] found that more frequent MP use recorded via a diary at baseline predicted higher depression scores on the Beck Depression Inventory (BDI) at one-year follow-up. Similarly, Liu et al. [8] found that baseline high MP use was associated with higher incidence of depressive and anxiety symptoms measured with the BDI and the Self-Rating Anxiety Scale (SAS) after eight months. However, two studies (both moderate risk) from the LIFE cohort did not find any association between baseline general MP use and internalising symptoms recorded via the Strengths & Difficulties Questionnaire (SDQ)—parent-reported [50] and self-reported [49] at one-year follow-up. This finding is consistent with the largest longitudinal study reviewed (low risk), a cohort study that found no association between baseline texting duration and depression or anxiety measured with the self-report versions of the Clinical Interview Schedule (CIS-R) in adolescents after two years [56].

*Externalising symptoms*: Three out of four studies (one low risk and two moderate risk) found a significant association between general use of MP/WD and measures of externalising symptoms. The first LIFE cohort study found that more frequent baseline parent-reported MP use predicted a higher score in the parent-reported hyperactivity/inattention and conduct problems SDQ subscales of young children after one year [50]. This evidence was consistent with the findings from two other studies: one found increase in conduct disorders after 18 months measured by ecological momentary assessment (EMA) [45] and the other found increase in concentration difficulties after one year measured by a four-point single-item Likert scale [48] in adolescents’ populations, both associated with more frequent self-reported texting [45, 48] and duration of MP calls [48]. The latter study also measured cumulative RF-EMF dose from MP/WD and far-field environmental sources and found that whole-body RF-EMF dose was associated with concentration difficulties when calculated from self-reported duration of use (duration of data traffic, cordless phones), but not when calculated from objective measures (network operator-measured data volume and call duration) [48]. The second LIFE cohort study found no significant association with baseline MP/WD usage and self-reported SDQ in adolescents at one-year follow-up [49].

*Wellbeing*: Two out of three studies (both moderate risk) found a significant association between general use of MP/WD and measures of wellbeing. Use of MP/WD over a school year was negatively associated with positive self-concept but not with general wellbeing in adolescents [55]. Conversely, Poulain et al. [49] found that adolescents with higher MP use at baseline reported a decrease in wellbeing measured with the health-related quality of life (HRQoL) scale by KIDSCREEN-27 at one-year follow-up. Another study (moderate risk) found that baseline duration of MP use for social communication had a positive indirect effect on children’s wellbeing measured with a bespoke scale at one and two-year follow-up, mediated through changes in social capital [57].

##### Cross-sectional findings

Twelve out of the 16 studies reporting cross-sectional findings included in this review examined associations between mental health outcomes and general use of MP/WD (Table 3). Two studies measured general use of MP and mental health, as well as problematic use of MP via specific questionnaires [46, 47], but as they did not report direct associations between duration or frequency of MP/WD use and mental health, we do not report their findings in this section.

*Internalising symptoms*: Six out of nine studies found significant cross-sectional positive associations between general use of MP/WD and measures of internalising symptoms [10, 11, 13, 15, 16, 52]. Most samples were adolescents and symptom measures varied from a single-item self-report to validated questionnaires. Overall, higher MP/WD use was associated with more anxiety or depressive symptoms, although in some studies this was limited to activities such as social networking and online chatting [11, 15] or in females only [12]. One study reported an association in the opposite direction, reporting that adolescents experienced less anxiety and depressive symptoms measured with the Multidimensional Anxiety Scale for Children (MASC) and BDI on days when sending more text messages [45]. Two studies did not find any significant association [51, 52].

One study also investigated the direct effect of RF-EMF on internalising symptoms [14], which showed that adolescents that used cordless phones had a higher likelihood of depressive symptoms compared to those who did not, but only true for cordless phones with frequencies ≤ 900 MHz [14].

*Externalising symptoms*: Three out of five cross-sectional studies found a significant positive association between general MP/WD use and measures of externalising symptoms (Table 3).

In particular, greater MP/WD use was related to concentration problems [16, 53], attention problems [16], hyperactivity symptoms [51], conduct problems [51], and hostility [15]. In contrast, no association was found with externalising symptoms reported by parents or teachers in young children [52].

*Wellbeing*: Two cross-sectional studies reported cross-sectional associations between general MP use and measures of wellbeing. One study found that adolescents who used MP for social media had significantly lower self-esteem [15]. Using more sophisticated modelling in a large sample of adolescents, Przybylski & Weinstein [59] described an inverted-U-shape relationship between digital-screen time and mental wellbeing, such that moderate engagement with MP is not harmful and may be advantageous, and effects may differ on weekdays compared to weekends.

#### Bedtime use of wireless devices

##### Longitudinal findings

Only one (low risk) out of 10 longitudinal studies included in this review examined associations between mental health outcomes and bedtime MP use, measured both at baseline and at three-year follow-up [60] (Table 4).

*Internalising symptoms*: Increased bedtime MP use from baseline to follow-up was not associated with changes in depressed mood measured with a bespoke 5-item scale, after adjusting for sleep behaviour [60].

*Externalising symptoms*: Increased bedtime MP use from baseline to follow-up was not associated with changes in externalizing behaviour measured with a bespoke 7-item scale, after adjusting for sleep behaviour [60].

*Wellbeing*: Increased bedtime MP use from baseline to follow-up was not associated with changes in coping abilities and self-esteem measured with bespoke 1 item and 3-item scales, after adjusting for sleep behaviour [60].

##### Cross-sectional findings

Four out of the 19 cross-sectional studies included in this review examined associations between mental health outcomes and bedtime MP/WD use (Table 2).

*Internalising symptoms*: All three studies investigating associations between bedtime MP use and measures of internalising symptoms found significant positive associations. In particular, more frequent and longer bedtime use was associated with higher depressive [58, 61], anxiety symptoms [58], suicidal feelings and self-injury [28]. However, in two studies this was partially mediated through reduced sleep duration [58] and sleep difficulties [61].

*Externalising symptoms*: No retrieved cross-sectional study investigated the associations between bedtime MP use and measures of externalising symptoms.

*Wellbeing*: One cross-sectional study described that adolescents who used MP at bedtime scored less on the HRQoL scale by KIDSCREEN-52 compared to those who did not, particularly when using screen mobile devices in a dark room [54].

## Discussion

This systematic review evaluated the current evidence on associations between MP/WD use and mental health outcomes in children and adolescents across 25 studies published up to 2019. With regards to our objectives, firstly, we found evidence to suggest that greater use of MP/WD may be associated with poorer mental health in children and adolescents, but that the strength of the associations vary partly depending on the time and nature of MP/WD usage. Secondly, we found evidence that bedtime MP/WD duration or frequency of use in particular is associated with worse mental health. Third, based on limited available research we found no evidence supporting a direct impact of RF-EMF on mental health. Finally, more studies are needed to clarify whether the different uses of MP/WD have distinct impacts on specific psychopathology. In particular, we found that the general use of MP/WD might be associated with externalising symptoms in children and adolescents.

We found substantial between-study heterogeneity in the choice of exposures and mental health outcomes, methods of exposure assessment, scales used to assess outcomes, study design, population selection, and approaches taken to address confounding variables—limiting our ability to infer general conclusions. This combined with the fact that a large proportion of studies (16 out of 25) were rated as high risk of bias, may explain the considerable between-study discrepancies on the presence/direction of associations found. Limitations to exposure assessment (as discussed below) imply that some associations could have been missed, while lack of correction for known confounding variables and differential recall bias in studies with cross-sectional design may have inflated the magnitude of associations [62]. Our synthesis is predominantly based on cross-sectional data, with few longitudinal studies to date producing inconsistent results.

The results of the current review largely align with recent systematic reviews on aggregated electronic screen time in children and young people, which have concluded that there are positive small but significant correlations between screen time and young children’s internalizing and externalizing behaviours [63, 64], and that longitudinal associations between screen time and depressive symptoms varied between different devices and uses [64].

### MP/WD usage

The strength and direction of associations between MP/WD use and mental health outcomes appear to depend on exposure-related factors including: the type of device, the purpose and the time-pattern of use, and the method of exposure assessment. For example, significant associations between MP use and symptoms of depression are reported for general MP use, but not when only measuring texting longitudinally [56] and fewer symptoms were reported on days when adolescents sent more texts in a cross-sectional study [45]. Similarly, no association with mental health outcomes emerged from specifically examining the effect of phone call duration or frequency in adolescents [14, 15, 48], unless calls occurred at night-time [58, 60]. Six studies specifically reported to be measuring smartphone use [11, 12, 15, 51, 57, 59]. Almost all other studies reported aggregated measures from devices capable of internet use with those that are not capable, making disentangling smartphone-specific effects impossible.

Overall, our observations are consistent with previous literature on differential effects depending on modes of technology use. For example, interactive screen time such as the use of a computer has been found to be more detrimental to sleep than passive screen time such as television watching [24, 65]. Historically, aggregated “screen time” was believed to impact health via displacing activity away from more adaptive behaviours [66], but this fails to capture the current diverse scopes of MP/WD use, from information seeking, to social interaction and entertainment [67]. Future studies should clarify how different modes of MP/WD use may have distinct psychological consequences, some of which are likely to foster resilience as well as increase vulnerability to mental health disorders.

An emerging area of the literature that holds promise explaining how the use of mobile phone use may explain variation in mental health in young people involves defining problematic mobile phone use or problematic smartphone use (PSU). This domain of behaviours has been conceptualised in a way that corresponds to the constructs of behavioural addiction. Previous studies have defined PSU through self-report scales with items with diagnostic criteria that resemble the criteria for substance use disorders (SUD), specifically symptoms of dependence such as loss of control (trouble limiting one’s smartphone use), tolerance (progressive increase in smartphone use to achieve the same psychological rewards) and withdrawal (negative symptoms on withdrawal) [68]. This approach has already shown that PSU is associated with poorer wellbeing and mental illness: a recent meta-analysis investigating psychological and behavioural dysfunctions related to smartphone use in young people has shown that PSU was associated with an increased odds of depression, anxiety, and stress; however, most research subjects within the pooled sample for depression and anxiety were over the age of 18 [18]. Furthermore, in common with other related constructs of problematic technology use associated with dysfunction (such as internet addiction and internet gaming addiction [69, 70]), some commentators have raised concerns that diagnosing individuals with PSU who display behavioural addictive symptoms with borrowed items from the diagnostic criteria of substance addiction disorders may not improve understanding of problematic use of technology’s aetiology and psychological sequelae [71, 72]. Nonetheless, although out the scope of this review, investigating MP/WD usage through the paradigm of PSU and addiction research with younger children, who are not yet as studied as college students, could potentially inform this field.

A major limitation in most studies was the choice of self-report measures to assess MP/WD exposure without external validation. Self-report device use is subject to measurement error such as recall difficulty and bias (e.g. call duration is considerably overestimated in adolescents populations [73, 74]). However, as children and adolescents favour online activity over calls and use wi-fi, data from self-report questionnaires may be more reliable indicators than activity inferred from operator-reported data [75]. Some self-report methods may be more robust, for example EMA may eliminate recall bias compared to self-report questionnaires or diaries [9], but participants may selectively respond to certain EMA signals [76]. Combining different methods of assessment has so far highlighted incongruencies [47, 48] and suggests a need for refining methodological rigour in measuring exposure. Future study-designs should confront these potential sources of bias by cross-validating different self-report instruments combined with device-recorded assessments of MP/WD use. Understanding measurement of MP/WD use and how likely exposure misclassification occurs is of critical importance. Some researchers have used duration of usage as a proxy for whether smartphone usage is problematic, i.e., is excessive and includes behaviours linked to addiction and impaired control. There is no established cut-off beyond which usage is defined as problematic, nor is usage alone sufficient for this classification without subjective distress [77]. Measures of problematic use can capture constructs that are distinct from measures of daily usage and duration, yet only with improving tracking and logging media use can the relationship between the two be understood [78].

### Assessment of mental health

Assessment of outcomes also included a wide range of different instruments, hindering direct comparison and limiting conclusive generalisable data synthesis. Mental health outcomes were investigated with a variety of self-report measures including ad hoc items [13, 16, 28, 53, 57], scales [8, 12, 17, 47, 49–52, 56, 59], sections of scales [9, 10, 14, 15, 45, 60, 61] and the same scale was even used with different cut-off levels [11, 12, 17, 46, 47, 49–52, 54, 55].

No study examined clinically diagnosed mental disorders and only one study used a self-report version of a structured interview: the CIS-R [56]. Given the public health relevance of this research area, we recommend use of validated instruments suitable for the general population but that have been standardised against clinical cut-offs (such as the PHQ-9, GAD-7, SDQ) and validated for younger children combined with parent-reported outcomes, such as the *Common Measures for Mental Health Science* [79]. Furthermore, robustness of findings would be increased by linkage with clinical data such as health records, with a view to drawing policy recommendations.

### Radiofrequency-EMF

We found no clear evidence supporting a direct effect of RF-EMF on mental health in children and adolescents. Only one study from the search time period directly assessed RF-EMF exposure using dosimeters [48]. Only designs that combine measures of device usage with measurements from all local RF-EMF sources (Bluetooth, other wireless networks), can discern whether RF-EMF dosage from MP/WD is responsible for variation in mental health outcomes. Even with these measures, disentangling effects is not straightforward, since MP/WD usage often co-occurs with changes in behaviour. Schoeni, et al. [48] showed one approach to addressing these issues; alongside self-reported device usage, network operator-reported calls and data traffic, and other local RF-EMF sources, they measured types of device usage deemed negative exposure controls for RF-EMF (gaming on computers, instant messaging). They found the duration of data traffic on the mobile phone, or the number of texts sent per day were more consistently associated with symptoms of concentration difficulties than one-year cumulative RF-EMF dose, suggesting mechanisms other than RF-EMF absorption were likely to explain differences in concentration. Further research in this area must move beyond exposimeter measurement and modelling of RF-EMF exposures given their inability to accurately measure RF levels from the user’s mobile phone. 5G base stations use narrow beams aimed from base stations to the user’s device. A large proportion of RF-EMF dosage will be triggered by a user’s device demanding data from the network, resulting in high spatio-temporal variations in the RF-EMF exposure. Future studies investigating effects of RF-EMF from mobile devices may now require personal exposure monitors worn on the body to address these challenges, whilst continuing to use device-reporting software, and activity-logging mapped with spatio-temporal data [80].

### The role of sleep

Consistent with previous literature, we found credible evidence that adverse outcomes may derive from MP/WD use at night. All cross-sectional studies examining bedtime use found a significant association with worse mental health, including higher levels of internalizing symptoms [28, 58, 61] and lower wellbeing [28, 54].

There is good evidence that sleep may act as a mediator for the effects of MP/WD on depression symptoms. Two studies found a mediating role for sleep difficulties [60, 61], and one found sleep duration mediated this relationship [58]. In both cases, the association between mobile phone use and depression was attenuated when conditioning on sleep and other demographic variables. This mediation could occur through the content of messages received, which could increase cognitive and emotional arousal [28, 60]. Alternatively, sleep quality could be affected by physical mechanisms such as melatonin suppression via exposure to bright light from screens, as observed in lab research [81, 82]; findings from Mireku et al. [54] support this as they found that young adolescents were found to have a greater likelihood of lower HRQoL when using MP/WD at night-time in the dark as opposed to with lights on. Two studies however found that the association between bedtime use and internalizing symptoms persisted even when adjusting for sleep duration [28, 58] or sleep latency [28]. Taken together, this suggests that MP/WD is only partially mediated by sleep duration or quality [26, 83], and may affect mental health through other mechanisms. Only one longitudinal study conditioned on sleep behaviour and found that the direct association between bedtime MP/WD and all mental health indicators was non-significant when controlling for sleep behaviour [60].

A few studies examining the effects of general MP/WD use also controlled for either sleep duration or sleep problems with mixed findings dependent on gender and purpose of device use [10, 12]. One study using cross-lagged panel analysis identified bidirectional longitudinal associations between both MP use and mental health outcomes as well as between MP use and sleep outcomes [8], suggesting that more complex models might be needed to infer the correct causal mechanisms.

Future longitudinal research should combine measures of both general and bedtime MP/WD use with sleep behaviour assessment. Given the known relationship between sleep disorders and behavioural problems such as delinquency, drug use and sexual risk-taking [84], future research should also investigate the role of sleep variables as potential confounders or mediators of the association between MP/WD use and externalising symptoms in children and early adolescents.

### Social media

We found that the association between MP and mental health outcomes was influenced by the nature and type of use, with social media more often associated with negative sequalae. Three cross-sectional studies found consistent evidence that social media use was associated with negative mental health outcomes in adolescents, in particular higher internalising symptoms including depression, anxiety, negative self-esteem and somatization [11, 12, 15] and also externalising symptoms [15]. More than two hours/day on social networking and online chats was associated with a higher risk of depression in Japanese adolescents, even when adjusting for sleep duration [11, 12]. These findings suggest that the content viewed or received, or the type of interactions developed by children and adolescents using MP/WD (e.g., on social media sites) may be harmful, rather than the duration of general use of MP/WD itself. Accordingly, recent research has focussed on potential harm from either broadcasted ideals driving feelings of inadequacy or social pressure to conform [85] or from normalising, triggering and contagion of harmful behaviour, such as self-harm [86] and orthorexia [87].

The specific relationship between social media and mental health outcomes may explain a chronological trend found in our review: only studies collecting data from 2012 onwards [8–12, 14–16] found a significant association between internalizing symptoms and general MP use in adolescents. In 2019, half the UK’s 10-year-olds own a smartphone, compared with only 18% of 8-11 s, and 62% of 12-15 s in 2012 [1, 88]. Smartphones allow truly mobile and continuous access to the internet, including at sensitive times (bedtime) and without parental supervision, which may explain this observed trend.

These initial findings need to be replicated in longitudinal studies dissecting the mental health impact of different types of MP/WD use. None of the reviewed studies probed for specific uses of social media (e.g., interpersonal support, social comparison), nor for the time spent on each platform. Unless the specific type of data and content viewed by children and adolescents on social media (and other online activities using MP/WD) is analysed, much of the commentary on the mechanism by which usage might affect mental health remains conjecture [89]. Digital phenotyping could represent a promising avenue towards understanding these mechanisms (as well as their interaction with other factors such as sleep). By measuring mental health symptoms and device-recorded children’s digital activities at a high temporal resolution [90], future studies could understand the relationship between inter-individual heterogeneity in mental health trajectories and MP/WD messaging patterns and online usage, supported by new technologies such as screenomics, the machine-learning assisted categorisation of images and text [91, 92]. Whilst there are ethical challenges, these could be overcome by collaborations between researchers and social media corporations (who already hold children’s social network activity data), as well as strong engagement work with young people and parents in co-producing acceptable frameworks for data capture, data protection and study design.

### Socio-demographic factors

Most but not all studies controlled for socio-demographic factors [15, 16, 45, 57] with considerable heterogeneity in the covariates included (e.g., age, gender, SES, parents’ education level, family composition and ethnicity). Many of these factors are known to be associated with both MP/WD use and mental health outcomes. For example, gender divides [93], and differences in households´ SES shift the use and access to information and communications technology [94], the pattern of use and how parents manage their teens’ technology use [95]. Failing to condition analyses on these variables, e.g. SES, is likely to exaggerate the relationship between MP/WD usage and mental health outcomes. Some studies reported that age and gender may modify the effect of MP/WD use on mental health, though findings were inconsistent [28, 61].

### When is MP/WD use positive for mental health?

A number of studies reported findings of a positive rather than detrimental association between MP/WD use and mental health [45, 57, 59]. Przybylski and Weinstein [59] describe a concave-down quadratic model that supports the Goldilocks Hypothesis, i.e. that moderate technology use is not harmful and even advantageous for wellbeing. A moderate MP/WD use for communication may strengthen social connections and provide access to support from interpersonal relationships and communities, which may, in turn, improve psychological wellbeing [96–98]. This is also supported by evidence that social capital mediates the effects of smartphone use for communication [57].

Understanding the positive impact of MP/WD use on children and adolescents’ mental health is crucial in the current context of the COVID-19 pandemic and related policy responses, such as physical distancing, social isolation, and school closures. Evidence from studies on online activity of adolescents from early phases of the pandemic (outside the scope of this review as they did not focus on MP/WD-specific behaviour) suggest that time fostering online connections could act as a buffer against the negative impact of isolation on mental health as online interactions are likely to mimic offline dynamics [99, 100]. However, other studies have found opposite findings: greater time on social media during the pandemic was related to higher depressive symptoms, despite lower feelings of loneliness [101], and divergent findings depending on the purpose of use and personality [102]. Given this dramatic change in context, we re-ran our search using our original search terms with the addition of COVID-19 keywords (see Supplementary Material). We found two studies that reported epidemiological analysis of child and adolescent MP/WD use and psychological outcomes during the pandemic compared to pre-pandemic assessments, but neither identified direct associations between post-pandemic change in MP/WD use and mental health [103, 104].

### Limitations

Due to heterogeneity in exposure and outcome assessments, we were not able to conduct a meta-analysis to calculate pooled effects. Device definitions in reviewed studies were often not specific, aggregating measures from devices capable of internet use with those that are not capable, making disentangling device- and activity-specific effects challenging. We encourage future reviews to conduct meta-analyses of specific MP/WD types of activity and their effects on mental health, as Sohn et al. have conducted with PSU [18]. A further consequence of not conducting a meta-analysis is that we were not able to estimate publication bias, evidence of which has been reported in a recent systematic review into child screen time and behaviour problems [63].

Our ability to infer causal relationships between MP/WD use and mental health was limited by the small number of longitudinal studies, and for those studies further limited by the assumption of unidirectional causal relationships. It remains unknown whether evidence on the effects technology use is skewed by children and adolescents seeking support for ongoing symptoms and bidirectional causal loops may exist between MP/WD usage and mental health [105, 106]. Indeed, a recent systematic review on longitudinal studies in this field reported the relation between screen time and subsequent depression was stronger than the reverse, i.e., depression and subsequent screen time [64].

Other limitations include: first, most evidence to date comes from high-income countries, which limits the generalizability of findings. Second, despite most of the studies controlling for SES, many studies relied on convenience samples drawn from schools instead of population-based samples and therefore may not reflect the global range of children’s social, cultural and economic environments. Third, we decided to adopt broad groupings of “internalizing symptoms”, “externalizing symptoms”, and “wellbeing” to synthesise the data, which may result in loss of important information about potential effects related to more specific disorders. Finally, although the role of cognitive function falls outside of the scope of the present review, it is well known that cognitive functioning affects emotional processing and therefore in turn mental health. This is particularly so in early adolescence when pubertal and cognitive development occur in tandem with radical changes in one’s social environment. Future research should investigate whether MP/WD use´s impact on cognitive function might mediate effects on mental health outcomes, and explore potential mechanistic pathways between MP/WD use, cognitive development and mental health.

### Conclusions and future directions

This systematic review expands upon previous work synthesizing findings regarding MP/WD usage and mental health from a predominantly under 18 years population. The studies included presented heterogeneous measures of both MP/WD usage and mental health, which limits the ability to synthesise findings in a conclusive and clinically meaningful way. More robust and standardised measures of MP/WD use are strongly needed to advance this area of research. In summary, we found suggestive evidence supporting a negative impact of general MP/WD use on externalising symptoms in children and early adolescents, while findings on internalising symptoms are less consistent. Sleep disturbance due to MP/WD use appears to influence mental health outcomes but the specific role of sleep remains to be clarified. Major gaps remain, such as the need to dissect effects based on different types of MP/WD use and in relation to specific population characteristics.

Despite the fears held around wireless technologies, we believe that at this stage there is not enough evidence supporting a causal negative relationship between MP/WD use and children and adolescent’s mental health to justify particular public health interventions. It is likely that a large between-subject variability exists in how MP/WD usage may predict the development of mental health outcomes based on the interaction with a child’s psychosocial context and neurobiological factors. Future research should focus on identifying groups at-risk for intervention or behavioural modification with respect to technology use. This is of increasing importance in the context of the COVID-19 pandemic, which is accelerating digital transformations and divides, including how much adolescents use technology for learning, connection and social support.

## Supplementary Information

Below is the link to the electronic supplementary material.

Supplementary file1 (DOCX 323 KB)

## Data Availability

Not applicable.
