# Supplementary material for: Impact of mobile phones and wireless devices use on children and adolescents’ mental health: a systematic review
Source: Eur Child Adolesc Psychiatry. 2022 Jun 16;33(6):1621–51. doi: 10.1007/s00787-022-02012-8 (PMC9200624; doi:10.1007/s00787-022-02012-8)
Supplement: Supplementary file 1 — Supplementary file1 (DOCX 323 KB) [file 787_2022_2012_MOESM1_ESM.docx]

**Table S1 – PRISMA checklist**

| **Section/topic** | **#** | **Checklist item** | **Reported on page** | |  |
| --- | --- | --- | --- | --- | --- |
| **TITLE** | | |  | |  |
| Title | 1 | Identify the report as a systematic review, meta-analysis, or both. | 1 | |  |
| **ABSTRACT** | | |  | |  |
| Structured summary | 2 | Provide a structured summary including, as applicable: background; objectives; data sources; study eligibility criteria, participants, and interventions; study appraisal and synthesis methods; results; limitations; conclusions and implications of key findings; systematic review registration number. | 2 | |  |
| **INTRODUCTION** | | |  | |  |
| Rationale | 3 | Describe the rationale for the review in the context of what is already known. | 4 | |  |
| Objectives | 4 | Provide an explicit statement of questions being addressed with reference to participants, interventions, comparisons, outcomes, and study design (PICOS). | 5,6 | |  |
| **METHODS** | | |  | |  |
| Protocol and registration | 5 | Indicate if a review protocol exists, if and where it can be accessed (e.g., Web address), and, if available, provide registration information including registration number. | 6 | |  |
| Eligibility criteria | 6 | Specify study characteristics (e.g., PICOS, length of follow-up) and report characteristics (e.g., years considered, language, publication status) used as criteria for eligibility, giving rationale. | 6 | |  |
| Information sources | 7 | Describe all information sources (e.g., databases with dates of coverage, contact with study authors to identify additional studies) in the search and date last searched. | 6 | |  |
| Search | 8 | Present full electronic search strategy for at least one database, including any limits used, such that it could be repeated. | 6 | |  |
| Study selection | 9 | State the process for selecting studies (i.e., screening, eligibility, included in systematic review, and, if applicable, included in the meta-analysis). | 6 | |  |
| Data collection process | 10 | Describe method of data extraction from reports (e.g., piloted forms, independently, in duplicate) and any processes for obtaining and confirming data from investigators. | 6,7 | |  |
| Data items | 11 | List and define all variables for which data were sought (e.g., PICOS, funding sources) and any assumptions and simplifications made. | 7 | |  |
| Risk of bias in individual studies | 12 | Describe methods used for assessing risk of bias of individual studies (including specification of whether this was done at the study or outcome level), and how this information is to be used in any data synthesis. | 7 | |  |
| Summary measures | 13 | State the principal summary measures (e.g., risk ratio, difference in means). | 7 | |  |
| Synthesis of results | 14 | Describe the methods of handling data and combining results of studies, if done, including measures of consistency (e.g., I^2^) for each meta-analysis. | 7 | |  |
| Risk of bias across studies | 15 | Specify any assessment of risk of bias that may affect the cumulative evidence (e.g., publication bias, selective reporting within studies). | 7 | |  |
| Additional analyses | 16 | Describe methods of additional analyses (e.g., sensitivity or subgroup analyses, meta-regression), if done, indicating which were pre-specified. | NA | |  |
| **RESULTS** | | | |  | |
| Study selection | 17 | Give numbers of studies screened, assessed for eligibility, and included in the review, with reasons for exclusions at each stage, ideally with a flow diagram. | | 7 | |
| Study characteristics | 18 | For each study, present characteristics for which data were extracted (e.g., study size, PICOS, follow-up period) and provide the citations. | | 7, 25-35 | |
| Risk of bias within studies | 19 | Present data on risk of bias of each study and, if available, any outcome level assessment (see item 12). | | 7 | |
| Results of individual studies | 20 | For all outcomes considered (benefits or harms), present, for each study: (a) simple summary data for each intervention group (b) effect estimates and confidence intervals, ideally with a forest plot. | | 7-11,25-35, 38 | |
| Synthesis of results | 21 | Present results of each meta-analysis done, including confidence intervals and measures of consistency. | | NA | |
| Risk of bias across studies | 22 | Present results of any assessment of risk of bias across studies (see Item 15). | | 8 | |
| Additional analysis | 23 | Give results of additional analyses, if done (e.g., sensitivity or subgroup analyses, meta-regression [see Item 16]). | | NA | |
| **DISCUSSION** | | | |  | |
| Summary of evidence | 24 | Summarize the main findings including the strength of evidence for each main outcome; consider their relevance to key groups (e.g., healthcare providers, users, and policy makers). | | 11-16 | |
| Limitations | 25 | Discuss limitations at study and outcome level (e.g., risk of bias), and at review-level (e.g., incomplete retrieval of identified research, reporting bias). | | 16,17 | |
| Conclusions | 26 | Provide a general interpretation of the results in the context of other evidence, and implications for future research. | | 17 | |
| **FUNDING** | | | |  | |
| Funding | 27 | Describe sources of funding for the systematic review and other support (e.g., supply of data); role of funders for the systematic review. | | 3 | |

**Table S2 – Detailed search strategy of the literature**

| **Data base** | **Search Strings** |
| --- | --- |
| Embase | (youth* or young or adolescen* or child* or teen*).mp.  *AND*  ((smart phone* or smartphone* or cell* phone* or (wireless or handheld or mobile or cordless)) adj1 (device* or tablet* or phone*)).mp.  *AND*  (psych* OR mental OR depress* OR anxi* OR stress* OR well-being OR wellbeing OR symptom*).mp. |
| MEDLINE | (youth* or young or adolescen* or child* or teen*).mp. or exp child/ or exp adolescent/ or exp adolescence/  *AND*  ((smart phone* or smartphone* or cell* phone* (wireless or handheld or mobile or cordless)) adj1 (device* or tablet* or phone*)).mp. or exp smartphone/ or exp mobile phone/  *AND*  (psych* or mental or depress* or anxi* or stress* or well-being or wellbeing or symptom*).mp. or exp mental health/ or exp mental stress/ or exp depression/ or exp anxiety/ or exp wellbeing/ |
| PsycINFO | (youth* or young or adolescen* or child* or teen*).mp. or exp Adolescent/ or exp Child/  *AND*  ((smart phone* or smartphone* or cell* phone* or (wireless or handheld or mobile or cordless)) adj1 (device* or tablet* or phone*)).mp. or exp Smartphone/ or exp Cell Phone/  *AND*  (psych* or mental or depress* or anxi* or stress* or well-being or wellbeing or symptom*).mp. or exp Psychiatry/ or exp Mental Health/ or exp Anxiety/ or exp Depression/ or exp Stress, Psychological/ |

.mp. = combined search fields (default if no fields are specified)

exp = “explodes” controlled vocabulary term (e.g., expands search to all more specific related terms in the vocabulary’s hierarchy)

**Table S3- Exposure characteristics of the included longitudinal studies**

| **Study Author(s)** | **Year** | **Study Design** | **Country** | **N** | **Age Range (Mean Age +/- SD)** | **% Female** | **Exposure devices** | **Purpose/Type of Use** | **Exposure Measure** | **Exposure time of day** | **Outcome Domain** | **Outcome** |
| --- | --- | --- | --- | --- | --- | --- | --- | --- | --- | --- | --- | --- |
| Babic et al. | 2017 | Longitudinal | Australia | 322 | 14.4  (0.6) | 65.5 | Tablet/mobile phone | Daily | Mean of weekday and weekend daily duration | Any | Internalising Externalising Well-being | SDQ  Other Well-Being Outcomes |
| Bae | 2019 | Longitudinal | South Korea | 2110 | 10.98  (0.18) | 48.5 | Smartphone | Daily | Frequency of smartphone use for communication only (SUFC) purposes | Any | Well-being | Subjective Well-being |
| Bickham et al. | 2015 | Longitudinal | United  States | 92 | 14.0 | 46.8 | Mobile phone | Daily | Mean of weekday and weekend daily duration | Any | Internalising | Depression |
| Calpbinici & Arslan | 2019 | Cross-sectional | Turkey | 426 | 16.05  (1.26) | 49.5 | Mobile phone | Calls and texting, social networking | Daily duration | Any | Internalising Externalising Well-being | Anxiety depression, other Externalizing Behaviours |
| Foerster & Röösli | 2017 | Cross-sectional | Switzerland | 412 | 14.1 | 48 | Mobile phone | Calls, text messages, surfing the Internet, social networking | Weekday and weekend daily duration | Any | Well-being | Other Well-Being Outcomes |
| George et al. | 2018 | Longitudinal | USA | 151 | 13.1  (0.91) | 48 | Mobile phone | texting | Daily duration every day of the week | Any | Externalising  Internalising (cross-sectional only) | Conduct Problem Behaviour |
| Guxens et al. | 2019 | Cross-sectional | Netherlands | 3102 | 5 | NR | Mobile phone, cordless landline phone | calls | Daily frequency | Any | Internalising Externalising | SDQ  Emotional/Peer Problems, Hyperactivity Problems, Behavioural Problems Subscale |
| Hosokawa & Katsura | 2018 | Cross-sectional | Japan | 1642 | 6.88 (0.35) | 48.8 | Smartphone, tablet | Overall use | Average daily duration on ‘typical day’ | Any | Internalising Externalising | SDQ  Emotional/Peer Problems, Hyperactivity Problems, Behavioural Problems Subscale |
| Ikeda and Nakamura | 2014 | Cross-sectional | Japan | 2698 | NR | 62.3 | Mobile phone | Overall use | Mean of weekday and weekend daily duration | Any | Internalising | Anxiety Depression |
| Khouja et al. | 2019 | Longitudinal | UK | 1869 | 16 | 49 | Mobile phone | Texting | Weekday and weekend daily duration | Any | Internalising | Anxiety Depression |
| Lemola et al. | 2015 | Cross-sectional | Switzerland | 362 | 14.8  (1.3) | 44.75 | Mobile phone | Calls, texting, surfing the Internet | Weekly frequency of use | Bedtime | Internalising | Depression |
| Liu et al. | 2018 | Longitudinal | China | 3396 | 18.3  (1.7) | 19.1 | Mobile phone | Overall use | Daily duration | Any | Internalising | Anxiety Depression |
| Mei et al. | 2019 | Cross-sectional | China | 3020 | NR | 49.21 | Mobile phone | Call, surfing the Internet, texting | Frequency of bedtime use | Bedtime | Internalising | Anxiety Depression |
| Mireku et al. | 2019 | Cross-sectional | UK | 6616 | M:12.1 (0.6)  F:12 (0.5) | 48.8 | Mobile phone, tablet, eBook reader, laptop | Overall use | Frequency of bedtime use | Bedtime | Well-being | Other Well-Being Outcomes |
| Mortazavi et al. | 2011 | Cross-sectional | Iran | 469 | 11  (2.33) | 49.89 | Mobile phone | Calls | Daily duration | Any | Internalising Externalising | Anxiety  ADHD symptoms |
| Nishida et al. | 2019 | Cross-sectional | Japan | 295 | 16.2 (0.9) | 41.4 | Smartphone | email, social networking, online chat, internet search, watching videos | Daily duration | Any | Internalising | Depression |
| Oshima et al. | 2012 | Cross-sectional | Japan | 17920 | EA: 13.7 (0.9)  LA: 16.6 (0.9) | 50.41 | Mobile phone | Overall use | Bedtime frequency | Bedtime | Internalising  Well-being | Suicidal Feelings  Self-Injury Other Well-Being Outcomes |
| Poulain et al. | 2018 | Longitudinal | Germany | 527 | 3.81  (0.89) | NR | Mobile phone | Overall use | Daily duration | Any | Internalising Externalising | SDQ subscales  (Emotional/Peer Problems, hyperactivity problems, behavioural problems) |
| Poulain et al. | 2019 | Longitudinal | Germany | 814 | 12.33 (1.67) | 50.9 | Mobile phone | Overall use | Daily duration | Any | Internalising Externalising  Well-being | SDQ subscales  (Emotional/Peer Problems, hyperactivity problems, behavioural problems), well-being |
| Przybylski & Weinstein | 2017 | Cross-sectional | England | 120,115 | 15 | NR | Smartphone | Social networking, chatting | Weekday and weekend daily duration | Any | Well-being | Depression |
| Redmayne et al. | 2013 | Cross-sectional | New Zealand | 373 | 12.3 | 44.2 | Mobile phone, cordless landline phone | Overall use | Average daily duration | Any | Internalising | Depression |
| Roser et al. | 2016 | Cross-sectional | Switzerland | 412 | 14 | 61.4 | Mobile phone | Calls, texting, data traffic | Daily frequency | Any | Internalising Externalising  Well-being | SDQ subscales  (Emotional/Peer Problems, hyperactivity problems, behavioural problems), other well-being outcomes |
| Schoeni et al. | 2017 | Longitudinal | Switzerland | 425 | 15.0  (0.79) | 59.8 | Mobile phone | Overall use | Average daily duration | Any | Externalising | ADHD symptoms |
| Tamura et al. | 2017 | Cross-sectional | Japan | 295 | 16.2 (0.9) | 41.4 | Smartphone | email, social networking sites, online chat, internet search, watching videos | Daily duration | Any | Internalising | Depression |
| Vernon et al. | 2018 | Longitudinal | Australia | 1101 | 13.5 | 57 | Mobile phone | Calls, texting | Average daily night-time duration | Bedtime | Internalising Externalising  Well- being | Depression, other externalizing behaviours, self-esteem,  self-concept, other well-being outcomes |
| NR: not reported; EA: Early Adolescents; LA: Late Adolescents | | | | | | | |  |  |  |  |  |

**Table S4 – Detailed Newcastle Ottawa Scale of each included longitudinal study**
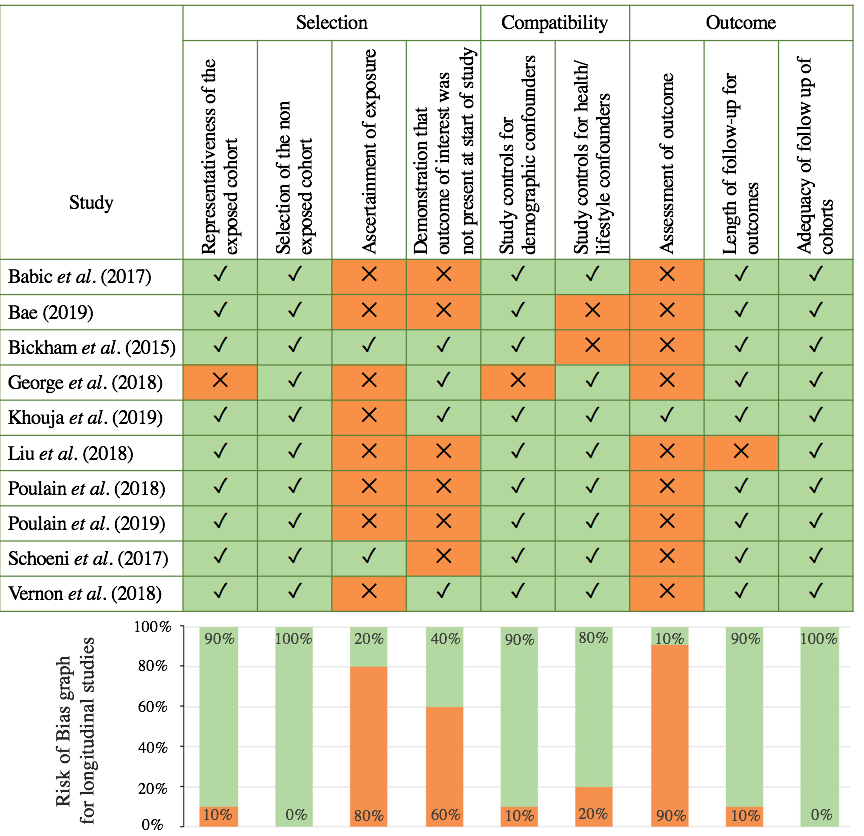


**Table S5 – Detailed Newcastle Ottawa Scale (adapted for cross-sectional studies) of each included cross-sectional study**


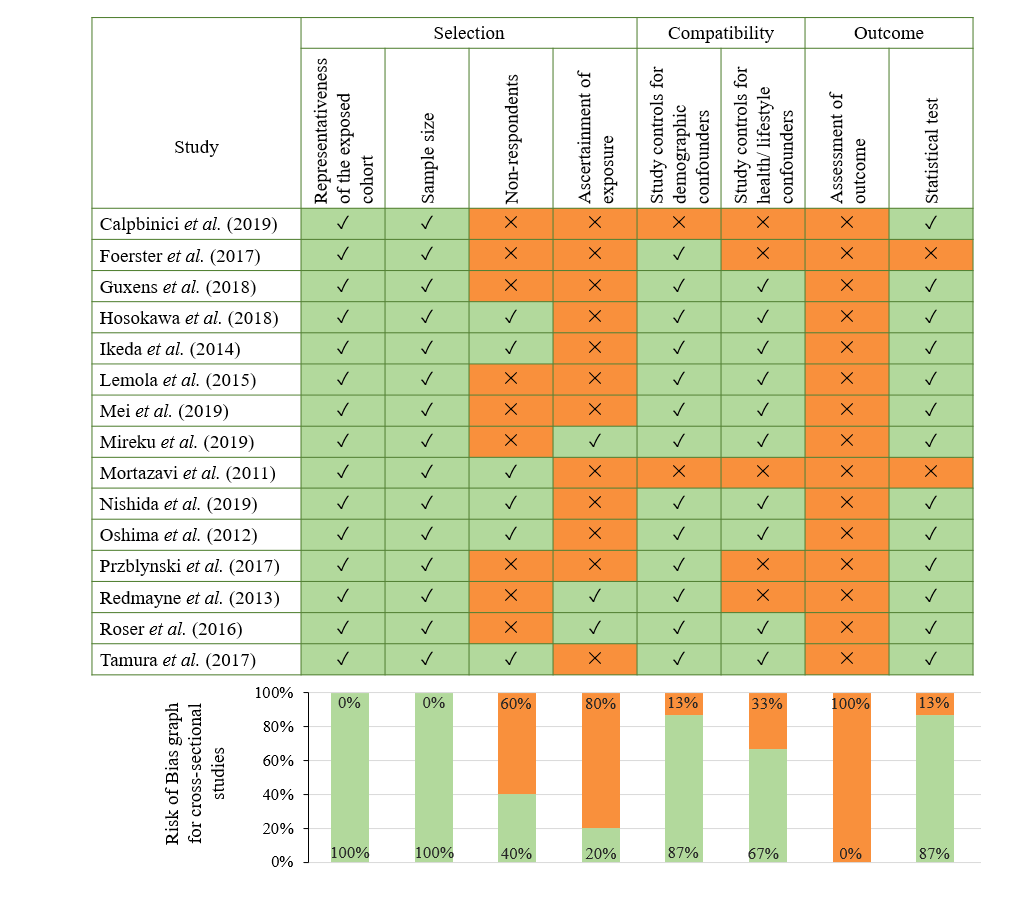


**Table S6 – Detailed Newcastle Ottawa Scales and AHRQ Conversions – Individual Longitudinal Studies**

| Study Author(s) | Year | Selection | | | | Compatibility | | Outcome | | | TOTAL | AHRQ Quality Band – Risk of Bias* |
| --- | --- | --- | --- | --- | --- | --- | --- | --- | --- | --- | --- | --- |
|  |  | **1** | **2** | **3** | **4** | **1a** | **1b** | **1** | **2** | **3** |  |  |
| Babic et al. | 2017 | ⭐ | ⭐ | - | - | ⭐ | ⭐ | - | ⭐ | ⭐ | 6 | Moderate |
| Bae | 2019 | ⭐ | ⭐ | - | - | ⭐ | - | - | ⭐ | ⭐ | 5 | Moderate |
| Bickham et al. | 2015 | ⭐ | ⭐ | ⭐ | ⭐ | ⭐ | - | - | ⭐ | ⭐ | 7 | Low |
| George et al. | 2018 | - | ⭐ | - | ⭐ | - | ⭐ | - | ⭐ | ⭐ | 5 | Moderate |
| Khouja et al. | 2019 | ⭐ | ⭐ | - | ⭐ | ⭐ | ⭐ | ⭐ | ⭐ | ⭐ | 8 | Low |
| Liu et al. | 2018 | ⭐ | ⭐ | - | - | ⭐ | ⭐ | - | - | ⭐ | 5 | High |
| Poulain et al. | 2018 | ⭐ | ⭐ | - | - | ⭐ | ⭐ | - | ⭐ | ⭐ | 6 | Moderate |
| Poulain et al. | 2019 | ⭐ | ⭐ | - | - | ⭐ | ⭐ | - | ⭐ | ⭐ | 6 | Moderate |
| Schoeni et al. | 2017 | ⭐ | ⭐ | ⭐ | - | ⭐ | ⭐ | - | ⭐ | ⭐ | 7 | Low |
| Vernon et al. | 2018 | ⭐ | ⭐ | - | ⭐ | ⭐ | ⭐ | - | ⭐ | ⭐ | 7 | Low |

**Key**: ⭐ = study meets NOS item criteria; ­- = study fails to meet NOS item criteria

***Categorization of Quality: Good quality** = 3 or 4 stars in selection domain AND 1 or 2 stars in comparability domain AND 2 or 3 stars in outcome/exposure domain; **Fair qualit**y = 2 stars in selection domain AND 1 or 2 stars in comparability domain AND 2 or 3 stars in outcome/exposure domain; **Poor quality** = 0 or 1 star in selection domain OR 0 stars in comparability domain OR 0 or 1 stars in outcome/exposure domain

**Table S7 – Detailed Newcastle Ottawa Scales and AHRQ Conversions – Individual Cross-Sectional Studies**

| Study Author(s) | Year | Selection | | | | Compatibility | | Outcome | | TOTAL | Study AHRQ Risk of Bias* |
| --- | --- | --- | --- | --- | --- | --- | --- | --- | --- | --- | --- |
|  |  | **1** | **2** | **4** | **4** | **1a** | **1b** | **1** | **2** |  |  |
| Calpbinici and Arslan | 2019 | ⭐ | ⭐ | - | - | - | - | - | ⭐ | 3 | High |
| Foerster and Röösli | 2017 | ⭐ | ⭐ | - | - | ⭐ | - | - | - | 3 | High |
| Guxens et al. | 2019 | ⭐ | ⭐ | - | - | ⭐ | ⭐ | - | ⭐ | 5 | High |
| Hosokawa and Katsura | 2018 | ⭐ | ⭐ | ⭐ | - | ⭐ | ⭐ | - | ⭐ | 6 | High |
| Ikeda and Nakamura | 2014 | ⭐ | ⭐ | ⭐ | - | ⭐ | ⭐ | - | ⭐ | 6 | High |
| Lemola et al. | 2015 | ⭐ | ⭐ | - | - | ⭐ | ⭐ | - | ⭐ | 5 | High |
| Mei et al. | 2019 | ⭐ | ⭐ | - | - | ⭐ | ⭐ | - | ⭐ | 5 | High |
| Mireku et al. | 2019 | ⭐ | ⭐ | - | ⭐ | ⭐ | ⭐ | - | ⭐ | 6 | High |
| Mortazavi et al. | 2011 | ⭐ | ⭐ | ⭐ | - | - | - | - | - | 3 | High |
| Nishida et al. | 2019 | ⭐ | ⭐ | ⭐ | - | ⭐ | ⭐ | - | ⭐ | 6 | High |
| Oshima et al. | 2012 | ⭐ | ⭐ | ⭐ | - | ⭐ | ⭐ | - | ⭐ | 6 | High |
| Przybylski and Weinstein | 2017 | ⭐ | ⭐ | - | - | ⭐ | - | - | ⭐ | 4 | High |
| Redmayne et al. | 2013 | ⭐ | ⭐ | - | ⭐ | ⭐ | - | - | ⭐ | 5 | High |
| Roser et al. | 2016 | ⭐ | ⭐ | - | ⭐ | ⭐ | ⭐ | - | ⭐ | 6 | High |
| Tamura et al. | 2017 | ⭐ | ⭐ | ⭐ | - | ⭐ | ⭐ | - | ⭐ | 6 | High |

**Key**: ⭐ = study meets NOS item criteria; ­- = study fails to meet NOS item criteria

***Categorization of Quality High Quality / Low Risk of Bias**: 3 or 4 stars in selection domain AND 1 or 2 stars in comparability domain AND 2 or 3 stars in outcome/exposure domain; **Fair quality / Moderate Risk of Bias**: 2 stars in selection domain AND 1 or 2 stars in comparability domain AND 2 or 3 stars in outcome/exposure domain; **Poor quality / High Risk of Bias**: 0 or 1 star in selection domain OR 0 stars in comparability domain OR 0 or 1 stars in outcome/exposure domain

**Table S8 - STROBE Statement Checklist of Items, individual studies**

| Author | Year | 1a | 1b | 2 | 3 | 4 | 5 | 6 | 7 | 8 | 9 | 10 | 11 | 12a | 12b | 12c | 12d | 12e | 13a | 13b | 13c | 14a | 14b | 14c | 15 | 16a | 16b | 16c | 17 | 18 | 19 | 20 | 21 | 22 |
| --- | --- | --- | --- | --- | --- | --- | --- | --- | --- | --- | --- | --- | --- | --- | --- | --- | --- | --- | --- | --- | --- | --- | --- | --- | --- | --- | --- | --- | --- | --- | --- | --- | --- | --- |
| Babic et al. | 2017 | **+** | **+** | **+** | **+** | **+** | **+** | **+** | **+** | **+** | **+** | **+** | **+** | **+** | **+** | **+** | **+** | **?** | **+** | **+** | **-** | **+** | **•** | **+** | **+** | **+** | **+** | **•** | **+** | **+** | **+** | **+** | **+** | **+** |
| Bae | 2019 | **+** | **+** | **+** | **+** | **+** | **+** | **+** | **+** | **+** | **-** | **+** | **+** | **+** | **-** | **-** | **+** | **-** | **+** | **+** | **-** | **?** | **-** | **+** | **+** | **+** | **•** | **•** | **+** | **+** | **+** | **+** | **+** | **+** |
| Bickham et al. | 2015 | **+** | **+** | **+** | **+** | **+** | **+** | **+** | **+** | **+** | **+** | **+** | **+** | **+** | **-** | **-** | **•** | **-** | **+** | **+** | **-** | **+** | **?** | **+** | **+** | **+** | **•** | **•** | **•** | **+** | **+** | **+** | **+** | **+** |
| Calpbinici and Arslan | 2019 | **+** | **+** | **+** | **+** | **+** | **+** | **+** | **+** | **+** | **-** | **+** | **+** | **+** | **-** | **-** | **+** | **•** | **+** | **+** | **-** | **+** | **+** | **•** | **+** | **+** | **+** | **•** | **+** | **+** | **-** | **+** | **+** | **+** |
| Foerster and Röösli | 2017 | **+** | **+** | **+** | **+** | **+** | **+** | **+** | **+** | **+** | **+** | **+** | **?** | **+** | **+** | **-** | **•** | **•** | **+** | **-** | **-** | **+** | **-** | **•** | **+** | **+** | **-** | **•** | **+** | **+** | **+** | **+** | **+** | **+** |
| George et al. | 2018 | **+** | **+** | **+** | **+** | **+** | **+** | **+** | **+** | **+** | **+** | **+** | **+** | **+** | **+** | **-** | **+** | **•** | **+** | **+** | **-** | **+** | **-** | **•** | **+** | **+** | **•** | **•** | **+** | **+** | **+** | **+** | **+** | **+** |
| Guxens et al. | 2019 | **+** | **+** | **+** | **+** | **+** | **+** | **+** | **+** | **+** | **+** | **+** | **+** | **+** | **•** | **+** | **+** | **•** | **+** | **+** | **+** | **+** | **+** | **•** | **+** | **+** | **+** | **•** | **+** | **+** | **+** | **+** | **+** | **+** |
| Hosokawa and Katsura | 2018 | **+** | **+** | **+** | **+** | **+** | **+** | **+** | **+** | **+** | **+** | **+** | **+** | **+** | **-** | **+** | **•** | **•** | **+** | **+** | **-** | **+** | **-** | **•** | **+** | **+** | **+** | **•** | **•** | **+** | **+** | **+** | **+** | **+** |
| Ikeda and •kamura | 2014 | **+** | **+** | **+** | **+** | **+** | **+** | **+** | **+** | **+** | **+** | **+** | **+** | **+** | **+** | **+** | **•** | **•** | **+** | **+** | **-** | **+** | **+** | **•** | **+** | **+** | **+** | **•** | **+** | **+** | **+** | **+** | **+** | **+** |
| Khouja et al. | 2019 | **+** | **+** | **+** | **+** | **+** | **+** | **+** | **+** | **+** | **+** | **+** | **+** | **+** | **+** | **+** |  |  |  |  |  |  |  |  |  |  |  |  |  |  |  |  |  |  |
| Lemola et al. | 2015 | **+** | **+** | **+** | **+** | **+** | **+** | **+** | **+** | **+** | **+** | **+** | **+** | **+** | **+** | **+** | **+** | **•** | **+** | **+** | **-** | **+** | **-** | **•** | **-** | **+** | **+** | **•** | **+** | **+** | **+** | **+** | **+** | **+** |
| Liu et al. | 2018 | **+** | **+** | **+** | **+** | **+** | **+** | **+** | **+** | **+** | **+** | **+** | **+** | **+** | **-** | **-** | **•** | **•** | **+** | **+** | **+** | **+** | **+** | **+** | **+** | **+** | **+** | **•** | **+** | **+** | **+** | **+** | **+** | **+** |
| Mei et al. | 2019 | **+** | **+** | **+** | **+** | **+** | **+** | **+** | **+** | **+** | **+** | **+** | **+** | **+** | **-** | **-** | **•** | **•** | **+** | **+** | **-** | **+** | **-** | **•** | **+** | **+** | **+** | **•** | **+** | **+** | **+** | **+** | **+** | **+** |
| Mireku et al. | 2019 | **+** | **+** | **+** | **+** | **+** | **+** | **+** | **+** | **+** | **+** | **+** | **+** | **+** | **+** | **+** | **•** | **+** | **+** | **+** | **-** | **+** | **+** | **•** | **+** | **+** | **+** | **•** | **+** | **+** | **+** | **+** | **+** | **+** |
| Mortazavi et al. | 2011 | **+** | **+** | **+** | **+** | **+** | **+** | **+** | **-** | **+** | **-** | **+** | **+** | **+** | **-** | **-** | **•** | **•** | **-** | **-** | **-** | **-** | **-** | **•** | **+** | **•** | **+** | **•** | **•** | **+** | **?** | **+** | **-** | **+** |
| Nishida et al. | 2019 | **+** | **+** | **+** | **+** | **+** | **+** | **+** | **+** | **+** | **+** | **+** | **+** | **+** | **+** | **+** | **+** | **•** | **+** | **+** | **-** | **+** | **+** | **•** | **+** | **+** | **+** | **•** | **+** | **+** | **+** | **+** | **+** | **+** |
| Oshima et al. | 2012 | **+** | **+** | **+** | **+** | **+** | **+** | **+** | **+** | **+** | **+** | **+** | **+** | **+** | **+** | **+** | **+** | **•** | **+** | **+** | **-** | **+** | **+** | **•** | **+** | **+** | **+** | **•** | **+** | **+** | **+** | **+** | **+** | **+** |
| Poulain et al. | 2018 | **+** | **+** | **+** | **+** | **+** | **+** | **+** | **+** | **+** | **+** | **+** | **+** | **+** | **+** | **-** | **+** | **+** | **+** | **+** | **-** | **+** | **+** | **+** | **+** | **+** | **+** | **•** | **•** | **+** | **+** | **+** | **+** | **+** |
| Poulain et al. | 2019 | **+** | **+** | **+** | **+** | **+** | **+** | **+** | **+** | **+** | **+** | **+** | **+** | **+** | **+** | **-** | **+** | **+** | **+** | **+** | **-** | **+** | **+** | **+** | **+** | **+** | **+** | **•** | **•** | **+** | **+** | **+** | **+** | **+** |
| Przybylski and Weinstein | 2017 | **+** | **+** | **+** | **+** | **+** | **+** | **+** | **+** | **+** | **+** | **+** | **+** | **+** | **•** | **-** | **+** | **•** | **+** | **+** | **-** | **-** | **-** | **•** | **+** | **+** | **+** | **•** | **+** | **+** | **+** | **+** | **+** | **+** |
| Redmayne et al. | 2013 | **+** | **+** | **+** | **+** | **+** | **+** | **+** | **+** | **+** | **+** | **+** | **+** | **+** | **-** | **+** | **+** | **•** | **+** | **+** | **-** | **+** | **+** | **•** | **+** | **+** | **+** | **•** | **•** | **+** | **+** | **+** | **+** | **+** |
| Roser et al. | 2016 | **+** | **+** | **+** | **+** | **+** | **+** | **+** | **+** | **+** | **+** | **+** | **+** | **+** | **+** | **+** | **•** | **+** | **+** | **+** | **-** | **+** | **+** | **+** | **?** | **+** | **+** | **•** | **+** | **+** | **+** | **+** | **+** | **+** |
| Schoeni et al. | 2017 | **+** | **+** | **+** | **+** | **+** | **+** | **+** | **+** | **+** | **+** | **+** | **+** | **+** | **+** | **+** | **+** | **+** | **+** | **+** | **-** | **+** | **+** | **+** | **+** | **+** | **+** | **•** | **+** | **+** | **+** | **+** | **+** | **+** |
| Tamura et al. | 2017 | **+** | **+** | **+** | **+** | **+** | **+** | **+** | **+** | **+** | **+** | **+** | **+** | **+** | **-** | **+** | **•** | **•** | **+** | **+** | **-** | **+** | **+** | **•** | **+** | **+** | **+** | **•** | **+** | **+** | **+** | **+** | **+** | **+** |
| Vernon et al. | 2018 | **+** | **+** | **+** | **+** | **+** | **+** | **+** | **+** | **+** | **+** | **+** | **+** | **+** | **+** | **+** | **+** | **•** | **+** | **?** | **-** | **+** | **+** | **+** | **+** | **+** | **+** | **•** | **+** | **+** | **+** | **+** | **+** | **+** |

Key: • = not applicable; + = item adequately reported; ­– = item not reported; ? = item unclear.

**NEWCASTLE - OTTAWA QUALITY ASSESSMENT SCALE COHORT STUDIES**

Note: A study can be awarded a maximum of one star for each numbered item within the Selection and Outcome categories. A maximum of two stars can be given for Comparability

**Selection**

1) Representativeness of the exposed cohort

a) truly representative of the average child/adolescent in the community **🟑**

b) somewhat representative of the average child/adolescent in the community **🟑**

c) selected group of users eg nurses, volunteers

d) no description of the derivation of the cohort

2) Selection of the non exposed cohort

a) drawn from the same community as the exposed cohort **🟑**

b) drawn from a different source

c) no description of the derivation of the non exposed cohort

3) Ascertainment of exposure

a) secure record (eg surgical records) measured from device or wireless network **🟑**

b) structured interview **🟑**

c) written self report

d) no description

4) Demonstration that outcome of interest was not present at start of study

a) yes **🟑**

b) no

**Comparability**

1) Comparability of cohorts on the basis of the design or analysis

a) study controls for gender or SES **🟑**

b) study controls for any pre-existing mental health condition **🟑**

**Outcome**

1) Assessment of outcome

a) independent blind assessment **🟑**

b) record linkage **🟑**

c) self report

d) no description

2) Was follow-up long enough for outcomes to occur

a) yes (select an adequate follow up period for outcome of interest) Select if more than 1 year **🟑**

b) no

3) Adequacy of follow up of cohorts

a) complete follow up - all subjects accounted for **🟑**

b) subjects lost to follow up unlikely to introduce bias - small number lost - > 75 % (select an adequate %) follow up, or description provided of those lost) **🟑**

c) follow up rate < 75 % (select an adequate %) and no description of those lost

d) no statement

**NEWCASTLE - OTTAWA QUALITY ASSESSMENT SCALE adapted for CROSS-SECTIONAL STUDIES**

Note: A study can be awarded a maximum of one star for each numbered item within the Selection and Outcome categories. A maximum of two stars can be given for Comparability

**Selection (Max = 4)**

1) Representativeness of the exposed cohort

a) truly representative of the average child/adolescent in the community **🟑**

b) somewhat representative of the average child/adolescent in the community **🟑**

c) selected group of users eg nurses, volunteers

d) no description of the derivation of the cohort

2) Sample Size

a) justified and satisfactory **🟑**

b) adequately powered to detect a difference (at least 10 events per variable in multivariate analyses)**🟑**

c) not justified

3) Non-repondents

a) comparability between respondents and non-respondents characteristics is established, and the response rate is satisfactory (>60%); or the response rate is excellent (>90%) **🟑**

b) the response rate is unsatisfactory, or the comparability between respondents and non-respondents is unsatisfactory

c) no description of the response rate or the characteristics of the responders and non-responders

4) Ascertainment of exposure

a) secure record (eg surgical records) measured from device or wireless network **🟑**

b) structured interview **🟑**

c) written self-report

d) no description

**Comparability (Max = 2)**

1) Comparability of cohorts on the basis of the design or analysis

a) study controls for gender or SES **🟑**

b) study controls for any pre-existing mental health condition **🟑**

**Outcome (Max = 2)**

1) Assessment of outcome

a) independent blind assessment **🟑**

b) record linkage **🟑**

c) self report

d) no description

2) Statistical test

a) The statistical test used to analyse the data is clearly described and appropriate, and the measurement of the association is presented as either an OR, CI and P value or a beta coefficient, SE and P value*****

b) The statistical test is not appropriate, not described or incomplete

**COVID-19 specific search-terms for post-hoc search** (Expert Search terms described at <https://tools.ovid.com/coronavirus/> [Accessed: 29^th^ January 2020])

**Ovid MEDLINE(R) All 1946 to present**

1. exp Coronavirus/
2. exp Coronavirus Infections/
3. (coronavirus* or corona virus* or OC43 or NL63 or 229E or HKU1 or HCoV* or ncov* or covid* or sars-cov* or sarscov* or Sars-coronavirus* or Severe Acute Respiratory Syndrome Coronavirus*).mp.
4. (or/1-3) and ((20191* or 202*).dp. or 20190101:20301231.(ep).) [this set is the sensitive/broad part of the search]
5. 4 not (SARS or SARS-CoV or MERS or MERS-CoV or Middle East respiratory syndrome or camel* or dromedar* or equine or coronary or coronal or covidence* or covidien or influenza virus or HIV or bovine or calves or TGEV or feline or porcine or BCoV or PED or PEDV or PDCoV or FIPV or FCoV or SADS-CoV or canine or CCov or zoonotic or avian influenza or H1N1 or H5N1 or H5N6 or IBV or murine corona*).mp. [line 5 removes noise in the search results]
6. ((pneumonia or covid* or coronavirus* or corona virus* or ncov* or 2019-ncov or sars*).mp. or exp pneumonia/) and Wuhan.mp.
7. (2019-ncov or ncov19 or ncov-19 or 2019-novel CoV or sars-cov2 or sars-cov-2 or sarscov2 or sarscov-2 or Sars-coronavirus2 or Sars-coronavirus-2 or SARS-like coronavirus* or coronavirus-19 or covid19 or covid-19 or covid 2019 or ((novel or new or nouveau) adj2 (CoV or nCoV or covid or coronavirus* or corona virus or Pandemi*2)) or ((covid or covid19 or covid-19) and pandemic*2) or (coronavirus* and pneumonia)).mp.
8. COVID-19.rx,px,ox. or severe acute respiratory syndrome coronavirus 2.os.
9. 6-8
10. 4 or 9

**Embase 1974 to present**

1. exp Coronavirus/
2. exp Coronavirus Infections/
3. (coronavirus* or corona virus* or OC43 or NL63 or 229E or HKU1 or HCoV* or ncov* or covid* or sars-cov* or sarscov* or Sars-coronavirus* or Severe Acute Respiratory Syndrome Coronavirus*).mp.
4. (or/1-3) and 20190101:20301231.(dc). [this set is the sensitive/broad part of the search]
5. 4 not (SARS or SARS-CoV or MERS or MERS-CoV or Middle East respiratory syndrome or camel* or dromedar* or equine or coronary or coronal or covidence* or covidien or influenza virus or HIV or bovine or calves or TGEV or feline or porcine or BCoV or PED or PEDV or PDCoV or FIPV or FCoV or SADS-CoV or canine or CCov or zoonotic or avian influenza or H1N1 or H5N1 or H5N6 or IBV or murine corona*).mp. [line 5 removes noise in the search results]
6. ((pneumonia or covid* or coronavirus* or corona virus* or ncov* or 2019-ncov or sars*).mp. or exp pneumonia/) and Wuhan.mp.
7. (coronavirus disease 2019 or 2019-ncov or ncov19 or ncov-19 or 2019-novel CoV or severe acute respiratory syndrome coronavirus 2 or sars-cov2 or sars-cov-2 or sarscov2 or sarscov-2 or Sars-coronavirus2 or Sars-coronavirus-2 or SARS-like coronavirus* or coronavirus-19 or covid19 or covid-19 or covid 2019 or ((novel or new or nouveau) adj2 (CoV or nCoV or covid or coronavirus* or corona virus or Pandemi*2)) or ((covid or covid19 or covid-19) and pandemic*2) or (coronavirus* and pneumonia)).mp.
8. (coronavirus disease 2019 or severe acute respiratory syndrome coronavirus 2).sh,dj.
9. 6-8
10. 4 and 9

**APA PsycINFO**

(coronavirus* or 2019-ncov or ncov19 or ncov-19 or 2019-novel Cov or ncov or covid or covid19 or covid-19 or covid 2019 or "coronavirus 2" or sars-cov2 or sars-cov-2 or sarscov2 or sarscov-2 or sars-coronavirus2 or sars-coronavirus-2 or SARS-like coronavirus* or coronavirus-19 or corona virus* or novel coronavirus*).mp

# **Glossary**

MB: Mobile Phone

MH: Mental Health

SDQ: Strengths and Difficulties Questionnaire.

WB: wellbeing

BMI:

SWB: Subjective Wellbeing

BDI: Beck Depression Inventory

EMA: Ecological Momentary Assessment

CD: Conduct Disorder

CIS-R: revised Clinical Interview Schedule

SAS: Self-Rating Anxiety Scale

BSI: Brief Symptom Inventory

HRQoL: Health-related quality of life

BDI: Beck Depression Inventory

CES-D: Center for Epidemiological Studies-Depression
